# A Degenerate Metal-Templated Catalytic System with Redundant Functional Groups for the Asymmetric Aldol Reaction

**DOI:** 10.1021/acs.joc.2c00414

**Published:** 2022-05-18

**Authors:** Alba Sors-Vendrell, Albert Ortiz, Diego Meneses, Ignacio Alfonso, Jordi Solà, Ciril Jimeno

**Affiliations:** Department of Biological Chemistry, Institute of Advanced Chemistry of Catalonia (IQAC−CSIC), Jordi Girona 18-26, Barcelona, Barcelona E08034, Spain

## Abstract

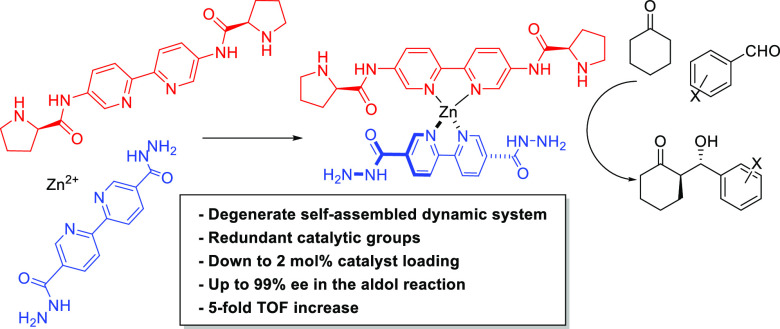

A degenerate zinc-templated catalytic system containing two bipyridine ligands with redundant functional groups for either enamine or hydrogen bond formation was applied to the asymmetric aldol reaction. This concept led to both a higher probability of reaction and rate acceleration. Thus, the catalyst loading could be decreased to a remarkable 2 mol % in what we think is a general approach.

Metal-templated catalysis is a powerful tool for reorganizing catalytic groups around a metal center, which provides structural support and the appropriate geometry for the catalytic event. It can be also viewed as organocatalysis through the coordination sphere of a metal complex.^1^ Many different reaction types can be catalyzed by such an approach,^2−14^ as the asymmetric aldol reaction is a fundamental C–C bond-forming protocol essential for increasing molecular complexity in organic synthesis.^15^ In particular, we are interested in organocatalyzed aldol reactions under bifunctional enamine/hydrogen bonding activation.^16−20^ Pioneering work by Meggers and co-workers showed that it is possible to catalyze an asymmetric α-amination reaction in an enamine/hydrogen bonding organocatalytic fashion by making use of an octahedral chiral-at-metal iridium complex.^21^

In contrast, we used a conceptually different approach consisting of a dynamic self-assembled system, wherein a mixture of catalytic ligands and a metal would generate a sufficient amount of the catalytically effective bifunctional species among other species in equilibrium of lesser, if any, catalytic importance.^22^ Therefore, we embarked on the development of a dynamic catalytic system for the asymmetric aldol reaction using two pyridine^16,17^ or two bipyridine^18^ ligands that contained either prolinamide (enamine-forming) or thiourea (hydrogen bonding) groups for the bifunctional catalysis. We used zinc or copper to assemble the tetrahedral complexes in a dynamic fashion. Several species can be present in the mixture, but the generation of any bifunctional kinetically competent complex was enough to make this approach successful.^18^ As additional advantages of this approach, the synthesis of the individual complexes is not required and the simple combination of the metal and ligands behaves as a catalytic system, reducing the synthetic cost.

At this point, we reasoned that doubling the number of functional groups per ligand would lead to the formation of a degenerate tetrahedral complex wherein chances of interaction between the two reacting substrates would increase even though identical catalytic groups were likely to be redundant and would not participate simultaneously in a given reaction (Figure 1). In this way, the reaction rate should also increase and catalyst loading could be reduced. Remarkably, biological degeneracy has been used to explain the relationship between robustness, complexity, and evolvability.^24^

**Figure 1:**
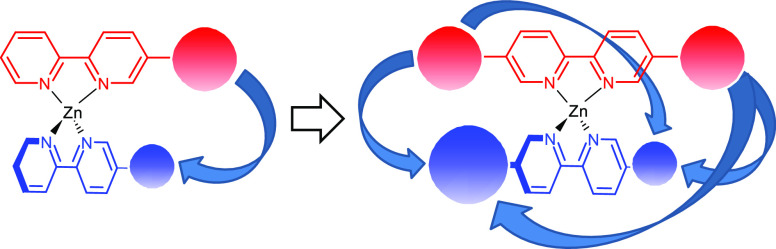
A degenerate complex with redundant functional groups increases the probability of reaction with substrates for bifunctional catalysis.

Despite the fact that physicochemical degeneracy is a slightly different concept, our systems also display some rudimentary complexity and adaptability. We reasoned that, as in biological systems, this unique combination of properties should lead to robustness, which would manifest as improved function.^22−24^

Following these ideas, herein we present the research that led us to identify new bipyridine ligands for the metal-templated highly asymmetric aldol reaction under low catalyst loadings. We synthesized the chiral 5,5′-bis(prolinamide)-2,2′-bipyridine ligand **9**, which we call **bipyPro**_**2**_, from the known 5,5′-diamino-2,2′-bipyridine (see the SI).^25,26^ Moreover, several intermediates with potential hydrogen bonding abilities are obtained along the route (diacid **2**, dihydrazide **6**, and dicarbamate **7**) or could be easily derived (dihydroxylamide **4** and diamide **5**, Figure 2). Experimental details and characterization data can be found in the SI.

**Figure 2:**
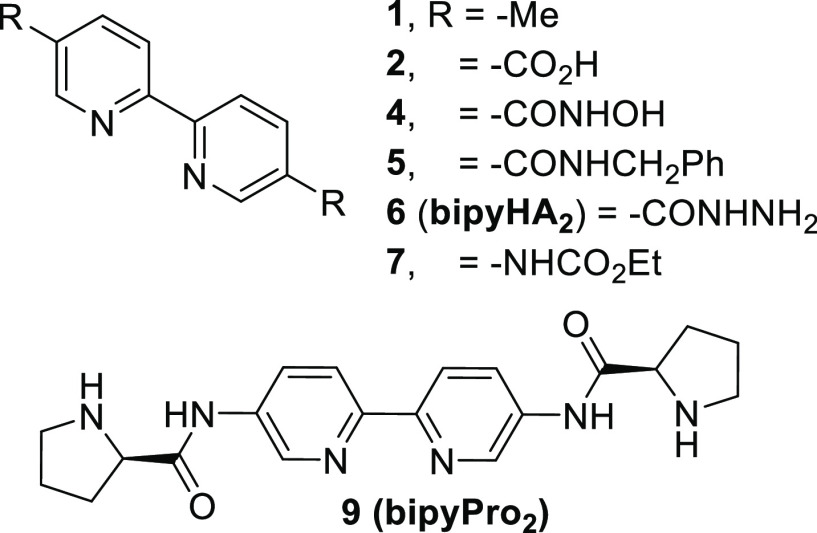
Enamine-forming ligand **9**; hydrogen-bonding ligands **2**, **4**, **5**, **6**, and **7**; and non-hydrogen-bonding ligand **1** used in this work.

Afterward, we decided to test these ligands in combination with the enamine-forming bipyridine **9** for metal-templated catalysis. The initial screening of hydrogen bonding ligands at 5 mol % catalyst loading using our previously developed conditions for the asymmetric aldol reaction between cyclohexanone and *p*-nitrobenzaldehyde^16,18^ are shown in Table 1. Zinc(II) trifluoroacetate was used as a template to furnish the desired catalytic systems.

**Table 1:**
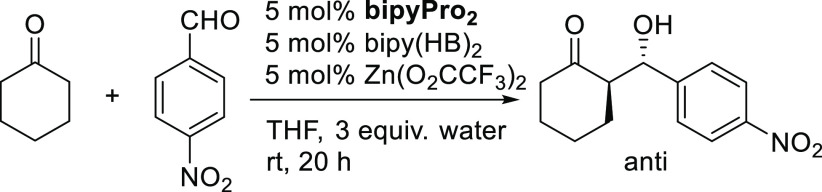
Screening of Hydrogen Bonding Ligands

entry	Bipy (HB)_2_	HB	conversion (%)^a^	d.r. (*anti/syn*)^a^	ee *anti* (%)^b^
1	**2**	–CO_2_H	78	14:1	97
2	**4**	–CONHOH	97	7:1	92
3	**5**	–CONHCH_2_Ph	88	6:1	95
4	**6**	–CONHNH_2_	>99	8:1	95
5^c^			>99^c^	14:1^c^	97^c^
6^d^			97^d^	14:1^d^	98^d^
7	**7**	–NHCO_2_Et	97	5:1	88
8	**1**	–CH_3_	99	6:1	89

a Determined by ^1^H NMR.

b Determined by chiral HPLC.

c Reaction run at 0 °C.

d Reaction run at 0 °C with 2 mol % catalyst loading.

To our delight, all hydrogen bonding ligands tested in Table 1 did actually produce good results, but a closer look revealed significant differences. Diacid **2** already provided a very high stereoselectivity, but reaction conversion was lower and the system was not fully soluble (entry 1). Hydroxyamide **4** yielded very good overall results (entry 2), whereas amide **5** gave a slightly higher ee but a lower conversion (entry 3). In contrast, dihydrazide **6** (called **bipyHA**_**2**_ hereafter) furnished a full conversion with a high ee (95%, entry 4) and was the consequent ligand of choice for the rest of this research. When the temperature was reduced to 0 °C, full conversion was still observed after 20 h, while the diastereo- and enantioselectivity improved (*anti/syn* 14:1 and 97% ee). The catalyst loading could be then reduced to 2 mol %, and very high conversion was conserved while the same degree of stereoselectivity was retained (98% ee, entry 6, Table 1). Dicarbamate **7** also furnished a high conversion, but the ee of the aldol product was lower than 90% (entry 7). Finally, a control experiment using non-hydrogen bonding bipyridine **1** rendered a very high conversion but a significantly lower enantioselectivity (89% ee, entry 8), highlighting the importance of the hydrogen bonding moiety for the control of the stereoselectivity. Other solvents were also tested, with poorer results than those of THF (see the SI).

At this point, it is worth pointing out how achiral bipyridine ligands are able to fine-tune the conversion and stereoselectivity of an organocatalytic bipyridine through metal templation in a process that resembles findings in asymmetric organometallic catalysis.^27^

Further experiments were carried out to assess the effects of the different building blocks of the catalytic system on the aldol reaction conversion (Figure 3). Free **bipyPro**_**2**_ catalyzed the reaction poorly, providing merely a 78% ee of the aldol product (black diamonds). The combination of **bipyPro**_**2**_ and Zn(II) increased the rate as well as the stereoselectivity to 92% ee (blue squares), likely due to a Lewis acid effect. When **bipyPro**_**2**_, **bipyHA**_**2**_, and Zn(II) were used together, reaction rate further increased, and 95% ee and full conversion were achieved in 10 h (Red triangles). Indeed, the determination of the apparent kinetic constants of these two last processes made it clear that the reaction containing the two ligands and zinc was roughly twice as fast as the reaction containing only **bipyPro**_**2**_ and Zn(II) (see SI).

**Figure 3:**
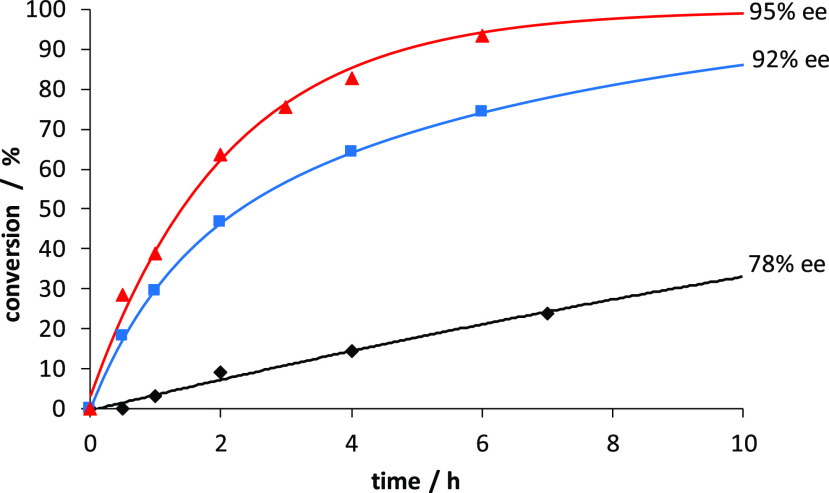
Asymmetric aldol reaction of cyclohexanone and *p*-nitrobenzaldehyde at rt catalyzed by 5 mol % **bipyPro**_**2**_, 5 mol % **bipyHA**_**2**_, and 5 mol % Zn(TFA)_2_ (95% ee, red triangles); 5 mol % **bipyPro**_**2**_ and 5 mol % Zn(TFA)_2_ (92% ee, blue squares); and 5 mol % **bipyPro**_**2**_ (78% ee, black diamonds).

Another set of control experiments was designed to ascertain the role of **bipyHA**_**2**_ (**6**) by substituting it for other ligands (Figure 4). Zinc-templated catalytic aldol reactions were performed using the non-hydrogen bonding ligand **1** instead of **6**; the reaction was slightly slower, and the ee was lower than 90% (black circles). A second experiment using twofold the amount of **bipyPro**_**2**_ (10 mol %) without **bipyHA**_**2**_ also exhibited diminished stereoselectivity (85% ee, empty red triangles and dotted red line), although the rate was essentially identical to that of the optimal catalytic system (bold red triangles and solid red line). Therefore, it appears that the second bipyridine ligand not only increases the reaction rate due to its sole presence in the system (likely due to the stabilization of the zinc complex) but also decisively modulates the stereoselectivity of the reaction. The observed improved rate and high ee in the presence of **bipyHA**_**2**_ must thus be attributed to the hydrazide group and its ability to form hydrogen bonds, similar to the other dicarboxylate derivatives **2**, **4**, and **5** from Table 1.

**Figure 4:**
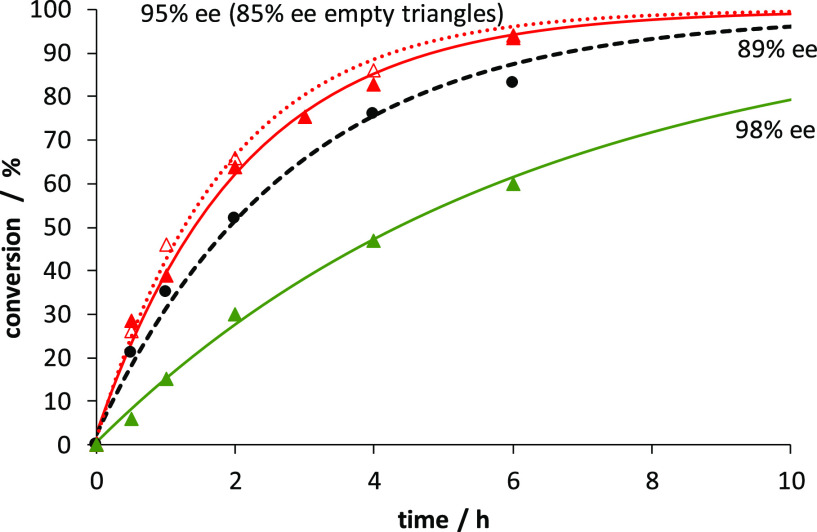
Asymmetric aldol reaction of cyclohexanone and *p*-nitrobenzaldehyde at rt catalyzed by 5 mol % **bipyPro**_**2**_, 5 mol % **bipyHA**_**2**_, and 5 mol % Zn(TFA)_2_ (95% ee, red bold triangles); 5 mol % **bipyPro**_**2**_, 5 mol % **1**, and 5 mol % Zn(TFA)_2_ (89% ee, black circles and dashed black line); 10 mol % **bipyPro**_**2**_ and 5 mol % Zn(TFA)_2_ (85% ee, red empty triangles and dotted red line); and 2 mol % **bipyPro**_**2**_, 2 mol % **bipyHA**_**2**_, and 2 mol % Zn(TFA)_2_ at 0 °C (98% ee, green triangles).

Finally, in Figure 4 we also plotted the evolution of the conversion with time for the optimized reaction at 0 °C using a 2 mol % catalyst loading (green triangles). The reaction is obviously slower, requiring 20 h to reach a conversion higher than 95%, as shown in Table 1.

As a final proof on the operation mode of the metal-templated system of **bipyPro**_**2**_, **bipyHA**_**2**_, and Zn(TFA)_2_, the catalyst order was determined and found to be 1 (see the SI).^28,29^ This result shows that only one assembly of the catalyst is responsible for the rate-determining step, further indicating the formation of a Zn–**bipyPro**_**2**_–**bipyHA**_**2**_ complex within the system, and that only one reaction takes place at a given time on the catalyst; this is, two aldol reactions do not happen simultaneously on the catalyst. Therefore, the functional groups of the catalyst are indeed redundant.

It could be argued that duplicating the functional groups of the catalyst should lead to a doubling of the catalytic activity. Even though that is not necessarily true (deleterious effects might appear just as with any structural modification), we calculated that the initial turnover frequency (TOF) based on the chiral bipyridine ligand concentration for the current catalytic system is five-times larger than that for the previous generation (see the SI for details). This is, the TOF does not increase twofold in the presence of double the amount of catalytic sites, but fivefold! This number is in good agreement with the fourfold increase in the probability of productive contacts for this degenerate bifunctional system with two redundant catalytic groups, as illustrated in Figure 1.

Finally, several aldehydes were tested as substrates for the asymmetric aldol reaction of cyclohexanone using a 2–5 mol % catalyst loading for Zn(TFA)_2_, **bipyHA**_**2**_**6**, and **bipyPro**_**2**_**9** (Table 2). Excellent results for both the yield and the stereoselectivity were obtained for electron-withdrawing aldehydes with 2 mol % catalyst loading (entries 1 and 2, Table 2). Less electron-withdrawing aldehydes required higher catalyst loading (5 mol %) to achieve reasonably good yields, but nevertheless excellent stereoselectivities were also obtained (entries 3–7). For example, *p*-chlorobenzaldehyde required three days of reaction to achieve a 75% yield, which led to some erosion of the stereoselectivity, but a remarkable 94% ee was still achieved (entry 3). To keep the very high diastereo- and enantioselectivities, the standard reaction time was used for the rest of the chloro-substituted benzaldehydes, which led to a slightly lower yields between 53 and 64% with great enantioselectivities (96–99% ee, entries 4–6). Clearly, *m*-fluorobenzaldehyde showed a better reactivity and stereoselectivity profile, and the corresponding aldol product could be isolated in a 75% yield and 98% ee (entry 7). In entry 8, we show results for sulfur-containing 4-oxothiane, which provided the product in an excellent yield and stereoselectivity at the 2 mol % catalyst loading. In contrast, non-six-membered ring ketones like cyclopentanone (entry 9) and acetone (entry 10) provided the corresponding aldol products in very modest diastereo- and enantioselectivities, albeit with very good reactivity, and the aldol product was isolated in a high yield.

**Table 2:**
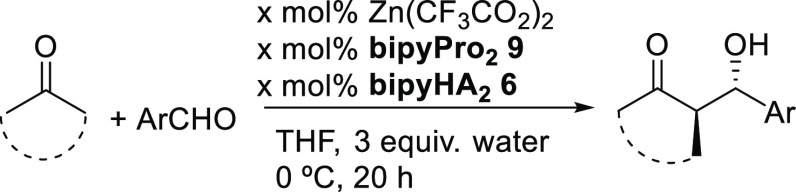

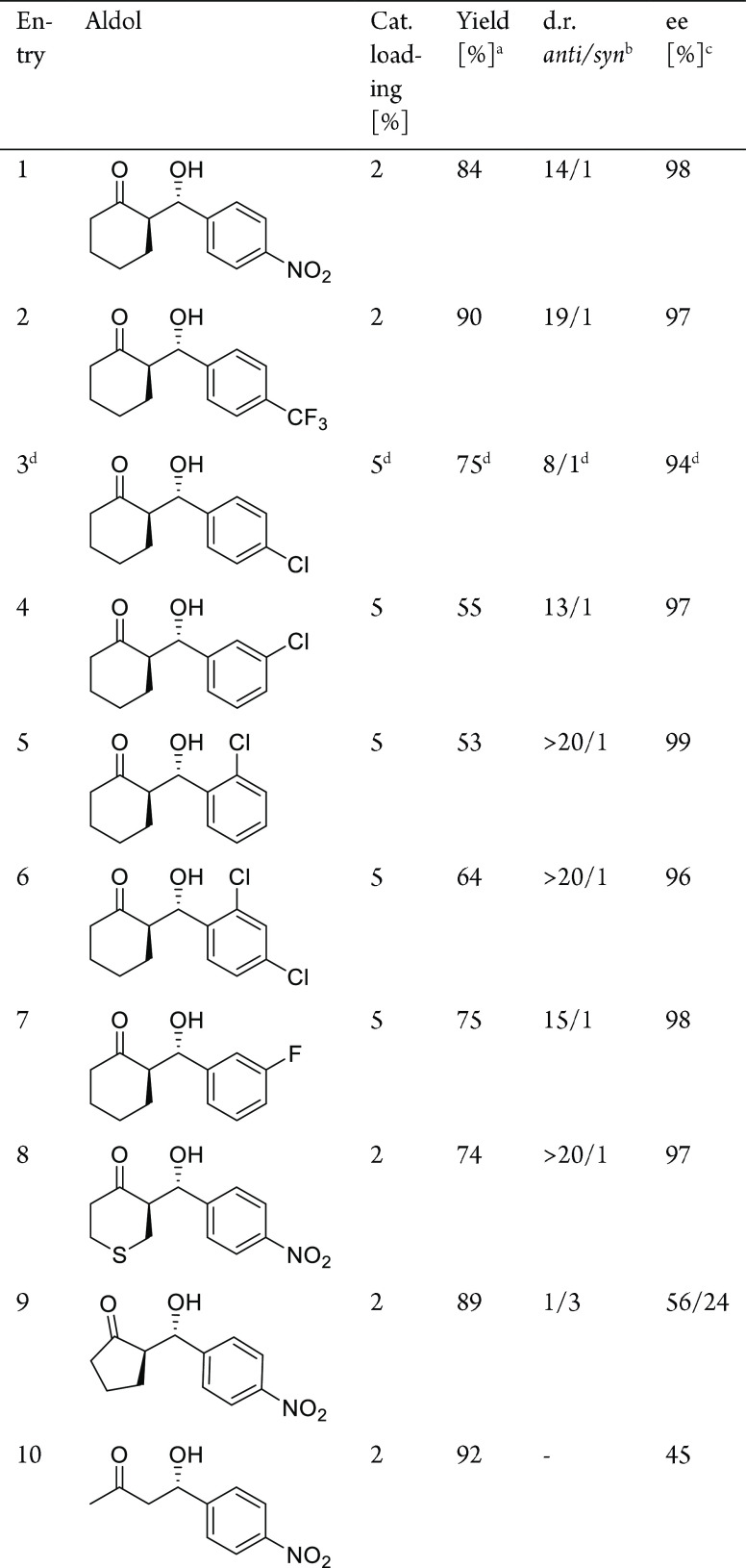
Substrate Scope

a Isolated yield.

b Determined by ^1^H NMR.

c Determined by chiral-phase HPLC.

d Reaction run for three days at a temperature from 0 °C to rt.

Regarding the catalyst loading, these results also represent a significant improvement (*vs* 5–10 mol % for our previous generation of catalysts).^18^

In conclusion, we have steadily developed two new ligands for metal-templated catalysis using the concepts of catalyst degeneracy and functional group redundancy. This approach has allowed us to improve both the reaction rate and the TOF and to reduce the catalyst loading accordingly. Indeed, loadings as low as 2 mol % have been successfully used for the asymmetric aldol reaction with electron-withdrawing aldehydes. Our results validate the concepts developed throughout this article as a useful design approach for enhancing the catalytic activity. Further work is in progress in our laboratory.

## Supporting Information Available

Experimental procedures, characterization data, NMR and FTIR spectra, HPLC traces, and mathematical treatments for the reactions analyses (PDF)

## Dedication

Dedicated to Prof. Antonio Delgado, *in memoriam*.
