# Supplementary material for: A Degenerate Metal-Templated Catalytic System with Redundant Functional Groups for the Asymmetric Aldol Reaction
Source: J Org Chem. 2022 May 18;87(11):7509–13. doi: 10.1021/acs.joc.2c00414 (PMC9171831; doi:10.1021/acs.joc.2c00414)
Supplement: Supplementary file 1 — jo2c00414_si_001.pdf [file jo2c00414_si_001.pdf]

SUPPORTING INFORMATION

FOR

A DEGENERATE METAL-TEMPLATED CATALYTIC SYSTEM WITH REDUNDANT FUNCTIONAL GROUPS FOR THE ASYMMETRIC ALDOL REACTION

Alba Sors-Vendrell, Albert Ortiz, Diego Meneses, Ignacio Alfonso, Jordi Solà* and Ciril Jimeno*

Institute of Advanced Chemistry of Catalonia (IQAC-CSIC), Jordi Girona 18-26, E08034 Barcelona (Spain)

ciril.jimeno@iqac.csic.es

jordi.sola@iqac.csic.es

1. Synthesis and characterization of di-5,5’-prolinamido-2,2’-bipyridine **9** S2
2. Synthesis and characterization of 2,2’-bipyridyl-5,5’-dihydroxamic acid **4** S7
3. Synthesis and characterization of 2,2’-bipyridyl-5,5’-dibenzylamide **5** S9
4. Spectra of ligands and intermediates S10
5. Procedure for the asymmetric aldol reaction and ee determination S17
6. Effect of solvents in conversion and stereoselectivity S18
7. Determination of apparent kinetic constants S19
8. Determination of catalyst order S20
9. Determination of catalyst TOF S22
10. Proposed reaction mechanism and origin of stereoselectivity S24
11. Transition state model figures S25
12. References S28

1. SYNTHESIS AND CHARACTERIZATION OF DI-5,5'-PROLINAMIDO-2,2'-BIPYRIDINE **9** (**bipyPro$_2$**)

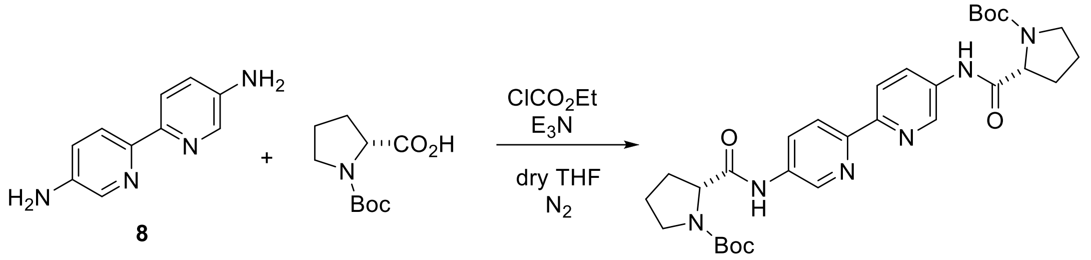

Scheme S1. Coupling between bipyridine **8** and Boc-proline.

In a flame-dried flask, D-Boc-proline (508.6 mg, 2.36 mmol, 2.2 eq) was dissolved in dry THF (8 mL) under N_2. At 0 ºC, Et_3N (600 µL, 4.30 mmol, 4 eq) and $ClCO_2Et$ (409 µL, 4.30 mmol, 4 eq) were successfully added. The mixture was stirred at 0 ºC for 30 minutes. Afterwards, the diaminobipyridine **8** was added (200 mg, 1.07 mmol, 1 eq) and the mixture further stirred at 0 ºC for 30 minutes, at rt for 2 h and at 70 ºC in an aluminum block overnight. After cooling to rt, the mixture was diluted with EtOAc, filtered and solvents were removed in a rotatory evaporator. The crude product was purified by silica gel column chromatography using a gradient of DCM (100 – 93 %) / MeOH (NH_3 1 N) (0 – 7 %) to give 200 mg of Boc-protected **9** (32% yield)

^{1}H-NMR (400 MHz, DMSO-d^6) δ 10.35 (s, 2H), 8.82 (dd, J = 12.5, 2.5 Hz, 2H), 8.28 (dd, J = 8.7, 3.9 Hz, 2H), 8.17 (dd, J = 8.7, 2.8 Hz, 2H), 4.36 – 4.17 (m, 2H), 4.17 – 4.01 (m, 2H), 3.52 – 3.33 (m, 2H), 2.29 – 2.10 (m, 2H), 1.91 – 1.79 (m, 6H), 1.40 – 1.27 (m, 18H) ppm.

HRMS (ESI-TOF) calcd. for $C_{30}H_{41}N_6O_6^+$ $[M+H]^+$ (m/z): 581.3082, found: 581.3085

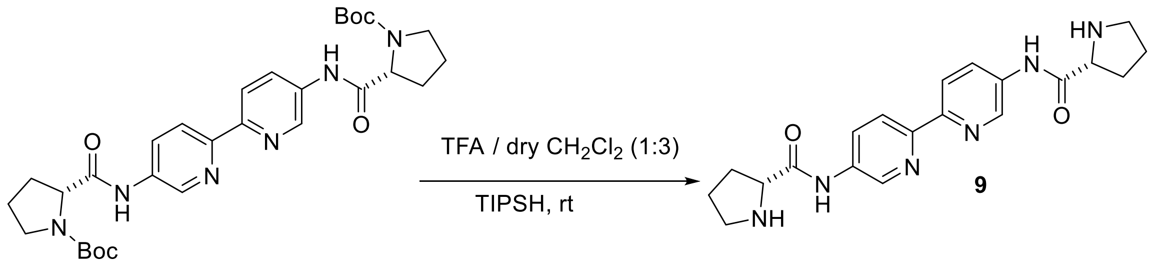

Scheme S2. Boc-group deprotection leading to ligand **bipyPro$_2$** (compound **9)**.

1.10 g (1.89 mmol, 1 eq) of Boc-protected **9** was dissolved in a mixture of dry DCM (11.34 mL) and TFA (3.78 mL). Then, 4.65 mL of triisopropylsilane were added and the mixture further stirred at rt for 2 h. Then, the liquids were evaporated and the resulting product was washed with Et_2O, dissolved with water and lyophilized. The obtained powder was purified by reversed-phase chromatography using a gradient of H_2O (100 – 0 %) – ACN (0 – 100%). The fraction containing the product was collected and the solvents were evaporated. Then, the obtained powder was dissolved in $CHCl_3$ and a solution of NaOH 2 M was added to make the solution weakly alkaline. Afterwards, the solution was extracted with $CHCl_3$ (3 × 50 mL) and the combined organic extracts were dried over anhydrous Na_2SO_4. The solvent was removed with a rotatory evaporator to afford 124 mg of **9** (56 % yield).

^{1}H NMR (400 MHz, $CDCl_3$) δ 9.94 (s, 2H), 8.70 (t, J = 1.6 Hz, 2H), 8.30 (d, J = 1.6 Hz, 4H), 3.90 (dd, J = 9.3, 5.2 Hz, 2H), 3.07 (ddt, J = 38.3, 10.3, 6.5 Hz, 4H), 2.34 – 1.96 (m, 4H), 1.91 – 1.72 (m, 10H), 1.40 – 1.22 (m, 2H) ppm.

$^{13}C\{^1H\}$ NMR (101 MHz, $CDCl_3$) δ 174.1 (Cq), 151.3 (Cq), 140.1 (CH), 134.4 (Cq), 126.4 (CH), 120.9 (CH), 61.0 (CH), 47.4 (CH_2), 30.8 (CH_2), 26.4 (CH_2) ppm.

HRMS (ESI-TOF) calcd. for $C_{20}H_{25}N_6O_2^+$ $[M+H]^+$ (m/z): 381.2034, found: 381.2018.

$[\alpha]_D^{25\,ºC}$ +79.2 (c=0.5, MeOH).

tert-butyl-(S)-2-((5’-((R)-1-(*tert*-butoxycarbonyl)pyrrolidine-2-carboxamido)-[2,2’-bipyridin]-5-yl)carbamoyl)pyrrolidine-1-carboxylate.

^{1}H NMR (400 MHz, DMSO-d^6):

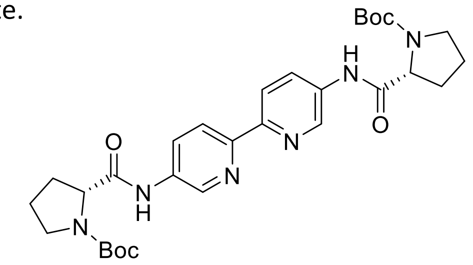

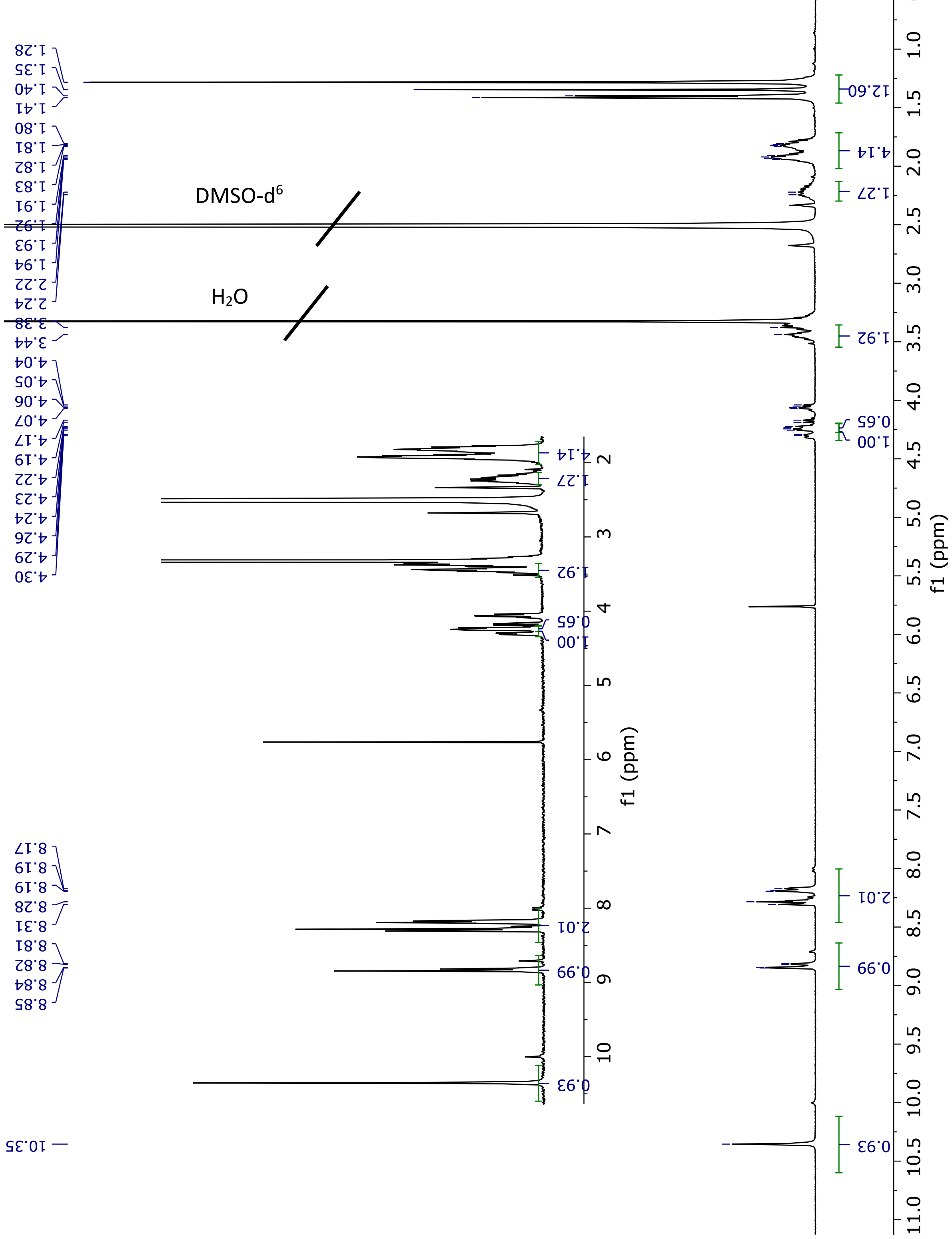

Figure S1. ^{1}H NMR spectrum of Boc-protected **bipyPro$_2$**.

di-5,5’-prolinamido-2,2’-bipyridine **9** (**bipyPro₂**).

^{1}H NMR (400 MHz, MeOD):

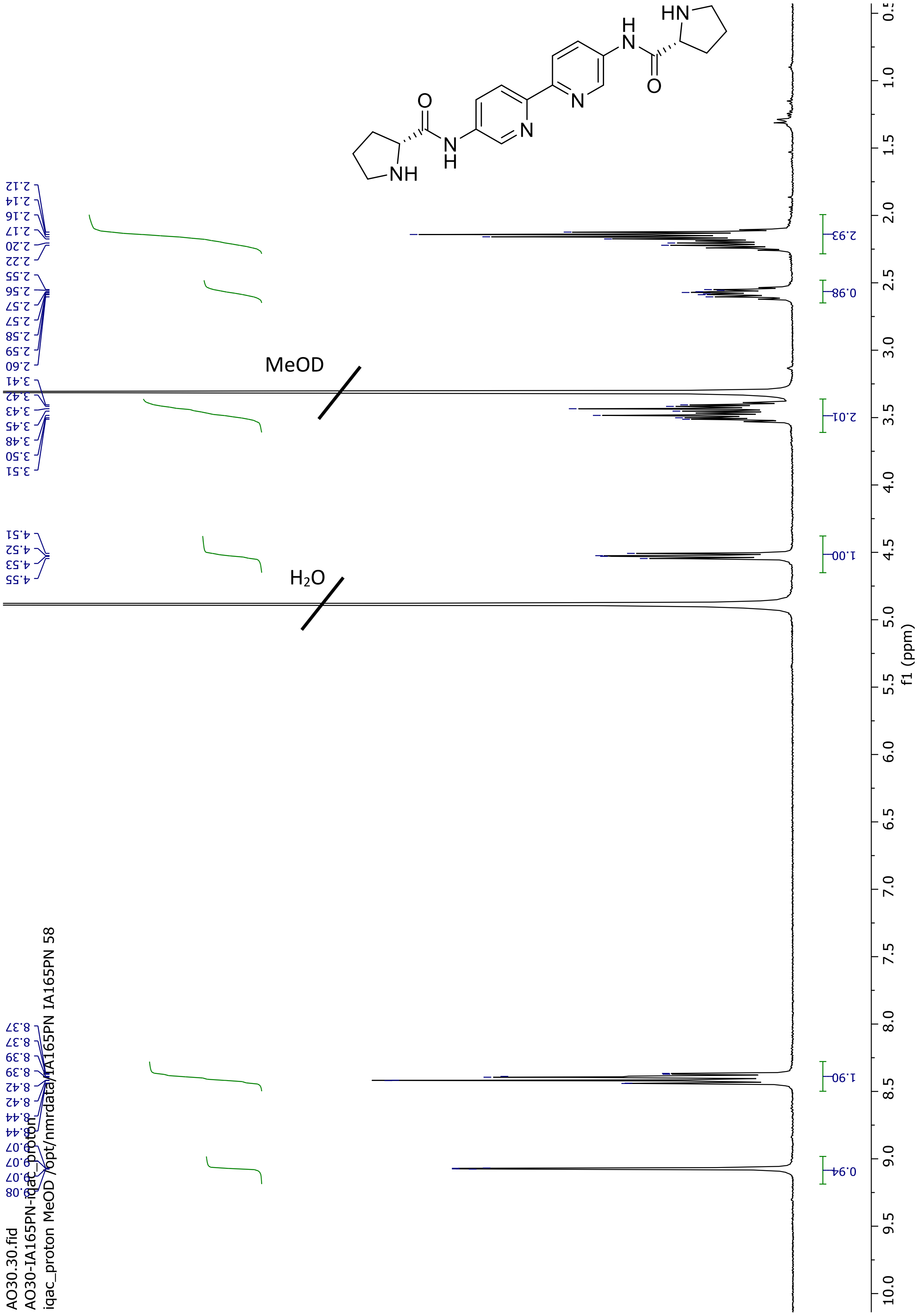

Figure S2. ^{1}H NMR spectrum of **bipyPro₂**.

di-5,5’-prolinamido-2,2’-bipyridine **9** (**bipyPro₂**).

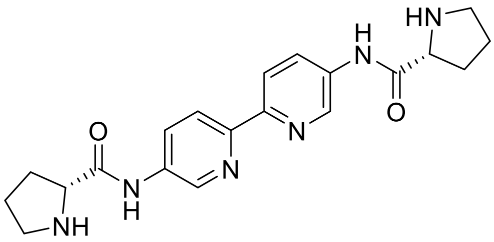

$^{13}C\{^{1}H\}$ NMR (100 MHz, $CDCl_3$):

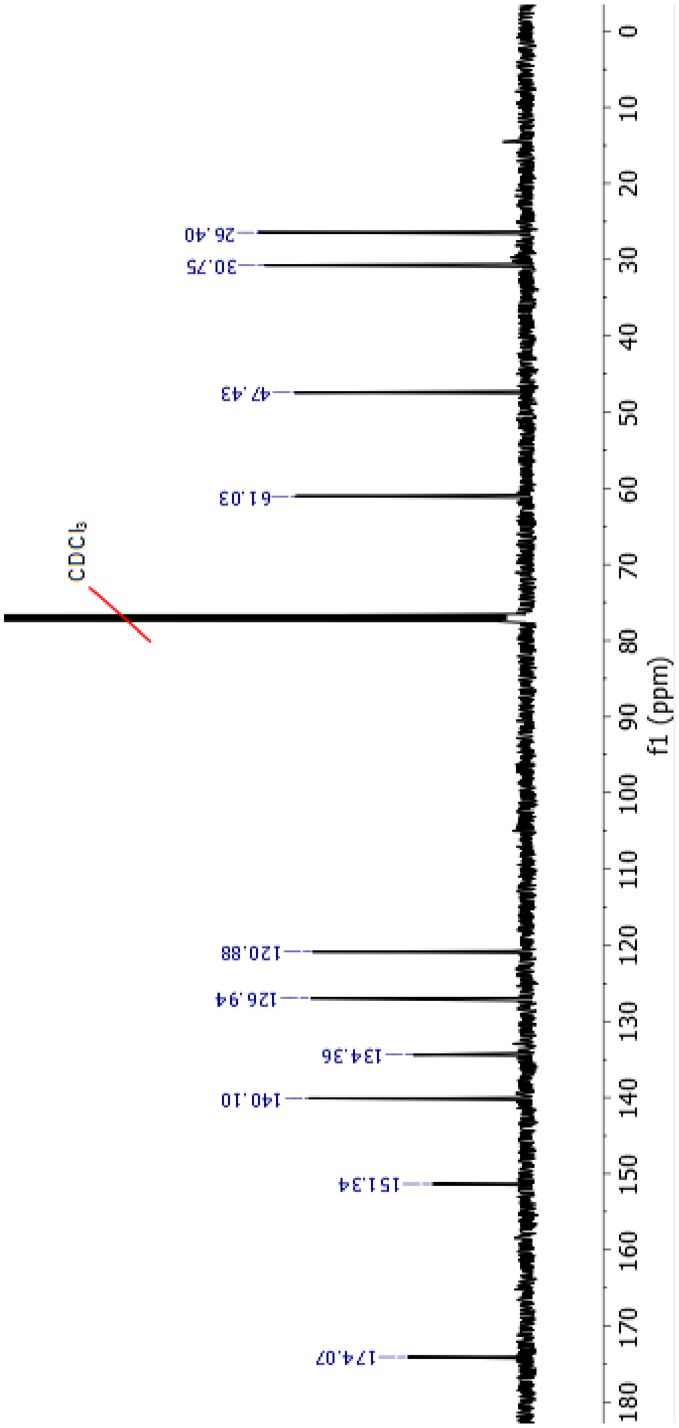

Figure S3. $^{13}C\{^{1}H\}$ NMR spectrum of **bipyPro₂**.

2. <u>SYNTHESIS AND CHARACTERIZATION OF 2,2'-BIPYRIDYL-5,5'-DIHYDROXAMIC ACID **4**</u>
   Procedure adapted from Eur. J. Med. Chem. 2019, 178, 116-130.[1]

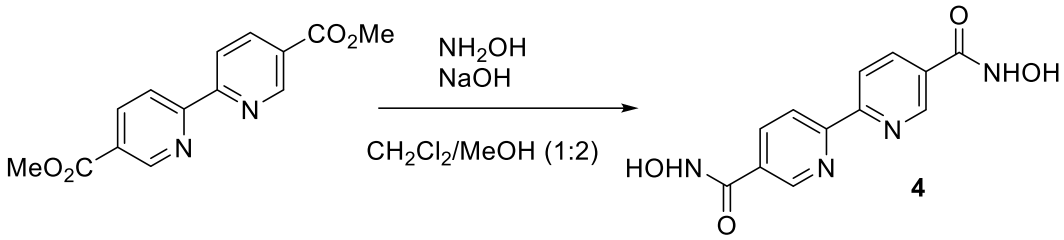

Scheme S3. Synthesis of dihydroxamic acid **4**.

2,2'-bipyridyl-5,5'-dicarboxylate methyl ester (272 mg, 1 mmol) was dissolved in 3 ml CH_2Cl_2 and 6 ml MeOH and cooled to 0 ºC. Then, hydroxylamine (4 ml 50% in water, 60 mmol) and sodium hydroxide (160 mg, 4 mmol) were added. The reaction was stirred at 0 ºC and left reacting overnight while warming up to rt. A bright yellow precipitate is formed. Ethanol was added and the mixture filtered. The precipitate was washed with ethanol and dried, furnishing 275 mg of dihydroxamic acid **4** (quantitative yield).

^1H NMR (400 MHz, DMSO-d^6) of **4**:

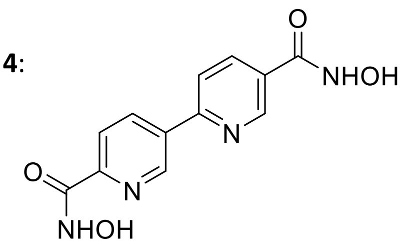

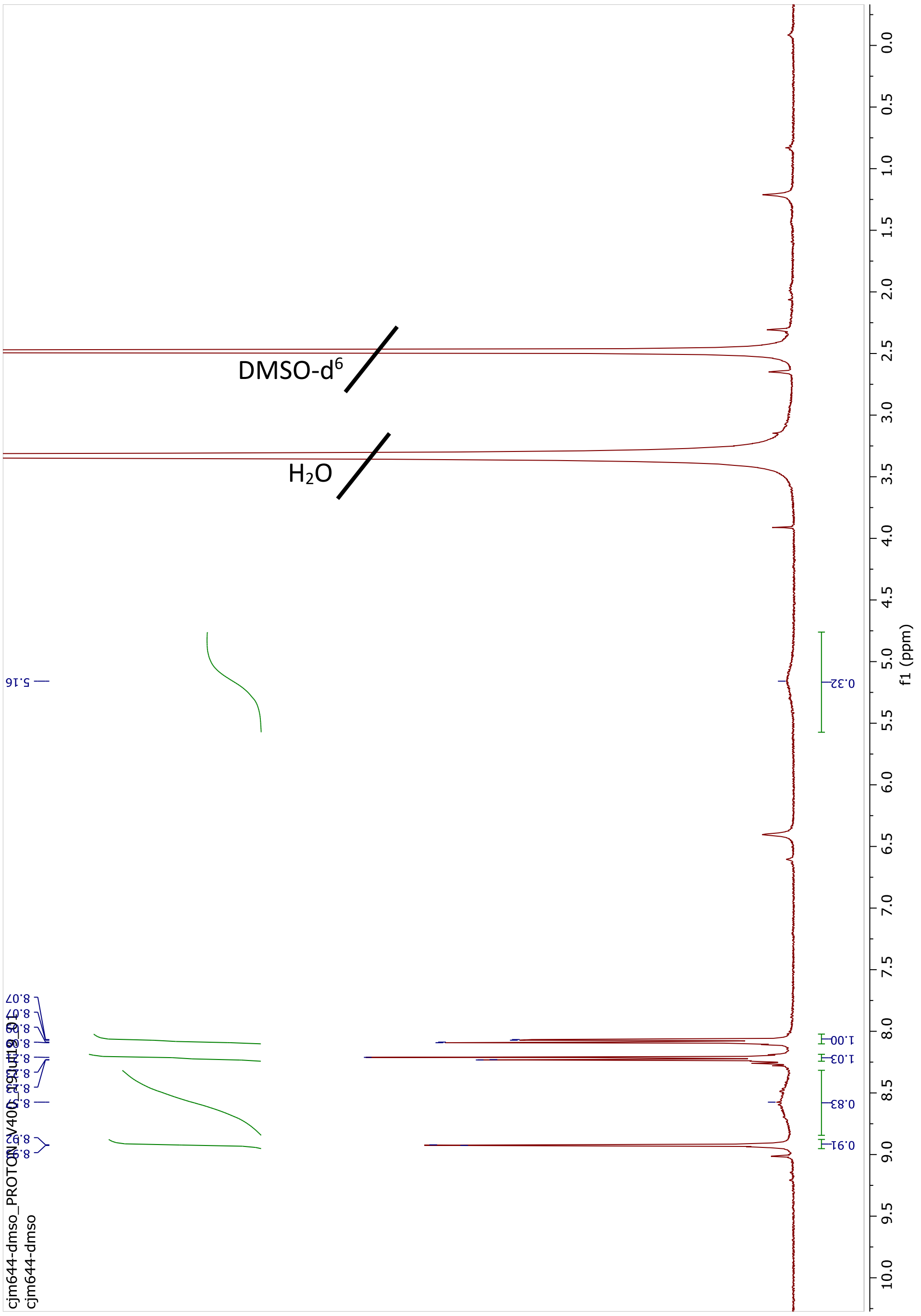

Figure S4. ^1H NMR spectrum of dihydroxamic acid **4**.

3. SYNTHESIS AND CHARACTERIZATION OF 2,2'-BIPYRIDYL-5,5'-DIBENZYLAMIDE **5**[2]

Described in ref. [2].

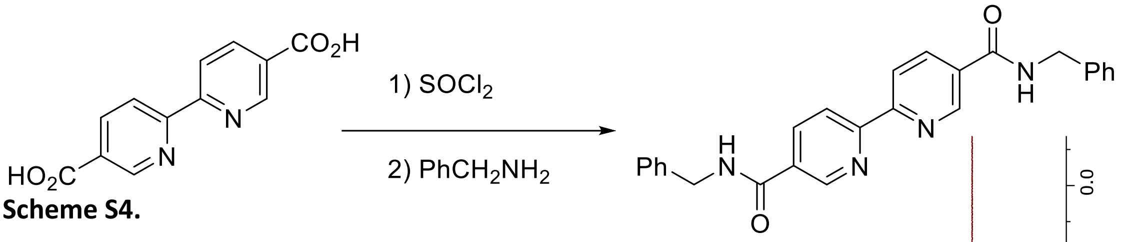

Scheme S4.

^{1}H NMR (400 MHz, DMSO-d^6) of **5**:

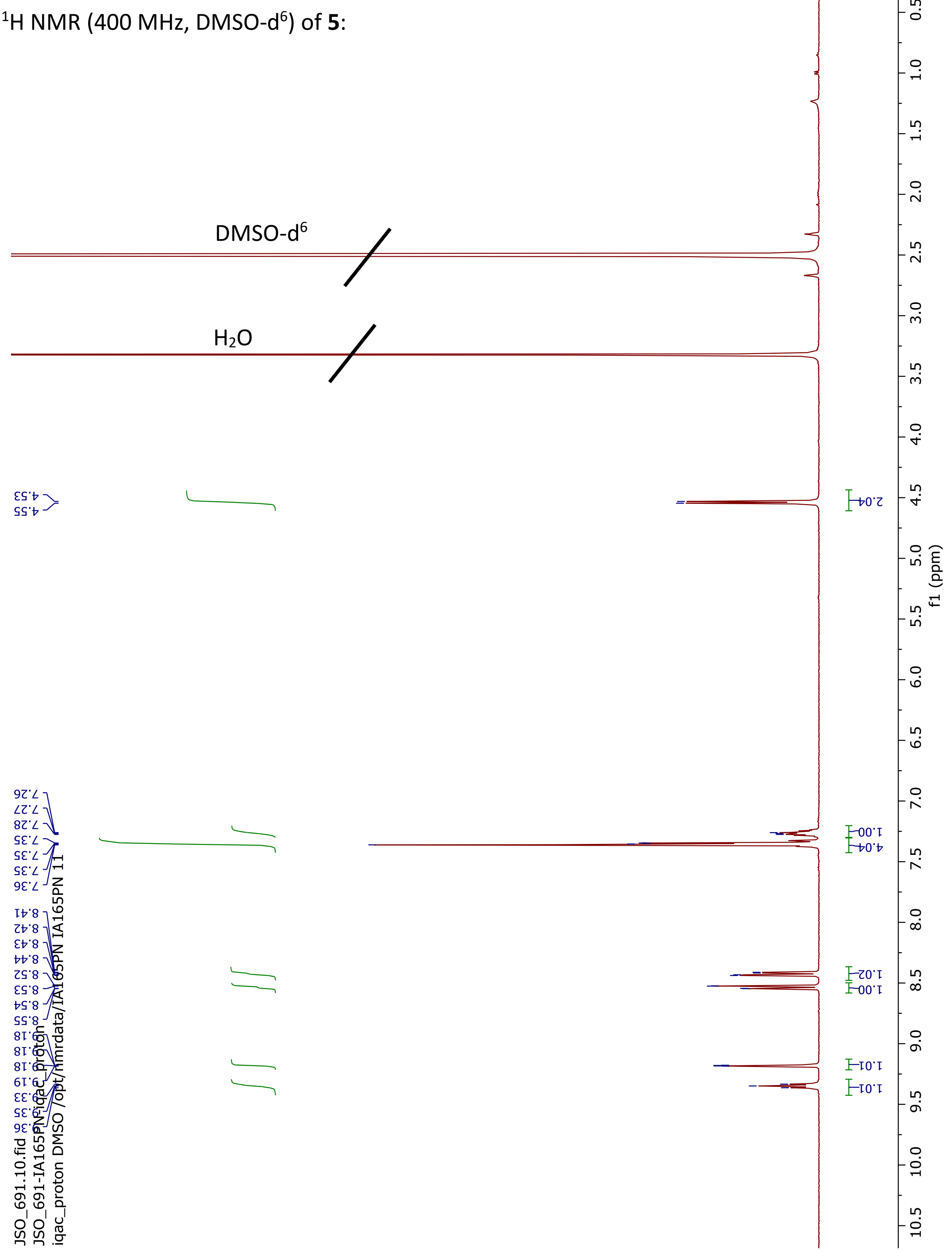

Figure S5. ^{1}H NMR spectrum of diamide **5**.

4. <u>SPECTRA OF LIGANDS AND INTERMEDIATES</u> [3]
Synthesized according to ref. [3]

2,2'-bipyridine-5,5'-dicarboxylic acid (**2**):

^{1}H NMR (400 MHz, DMSO-d^{6}):

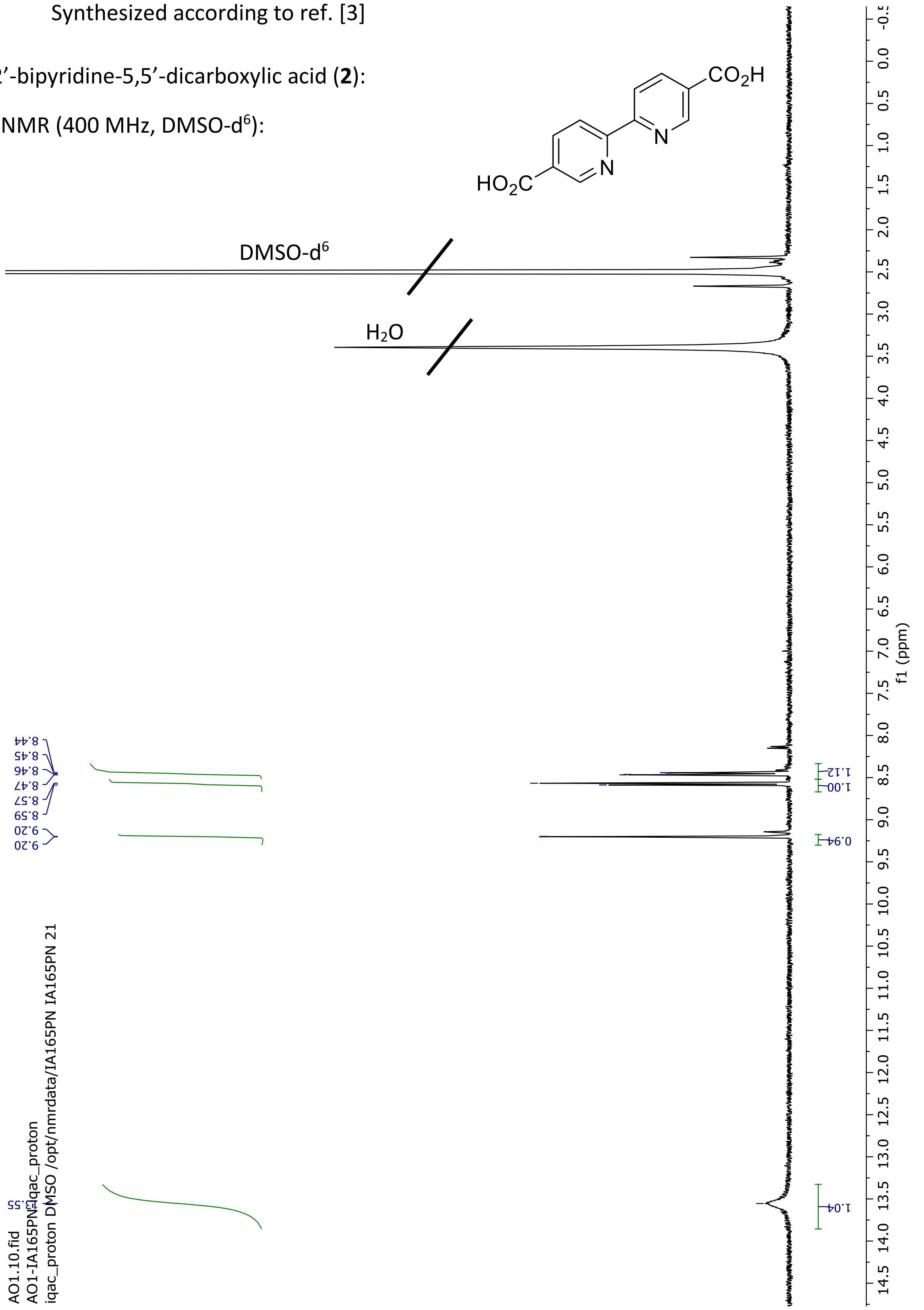

Figure S6. ^{1}H NMR spectrum of dicarboxylic acid **2**.

Diethyl 2,2'-bipyridine-5,5'-dicarboxylale (**3**):

^{1}H NMR (400 MHz, DMSO-d^6):

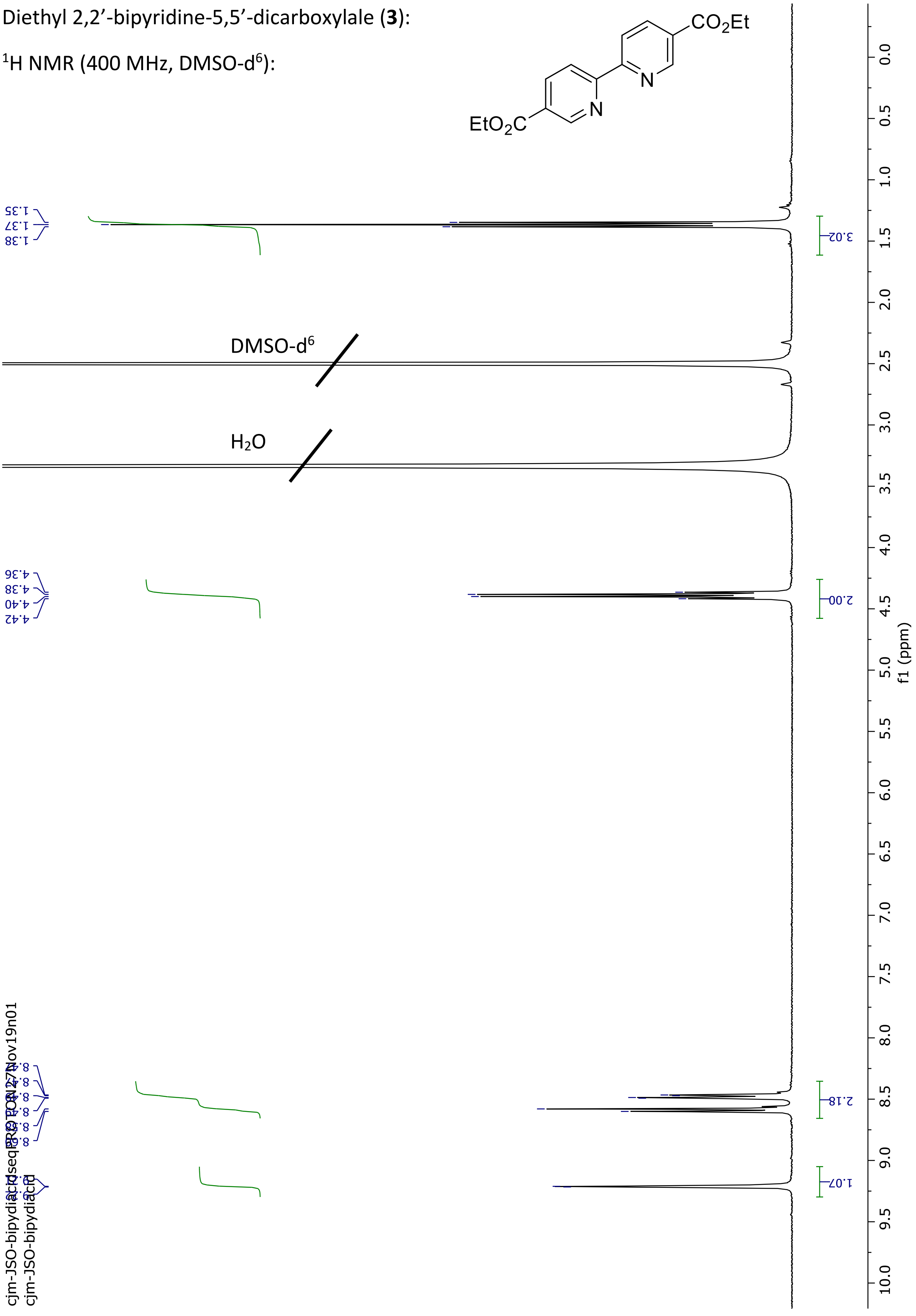

Figure S7. ^{1}H NMR spectrum of diester **3**.

2,2’-bipyridine-5,5’-dicarbohydrazide (**6**).

^{1}H NMR (400 MHz, DMSO-d^6) of **6**:

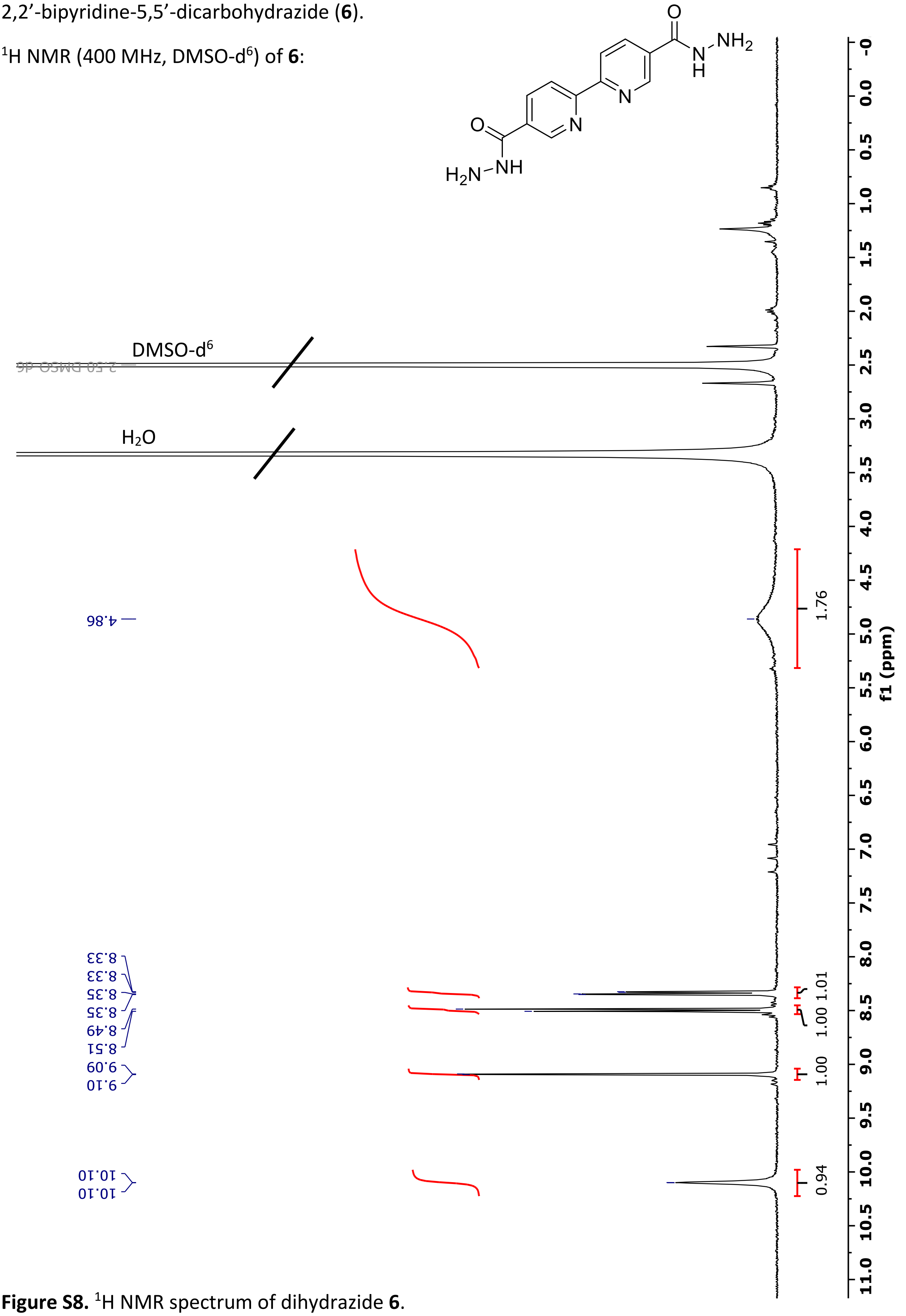

Figure S8. ^{1}H NMR spectrum of dihydrazide **6**.

2,2’-bipyridine-5,5’-dicarbonyl diazide (**8**).

^{1}H NMR (400 MHz, DMSO-d^6):

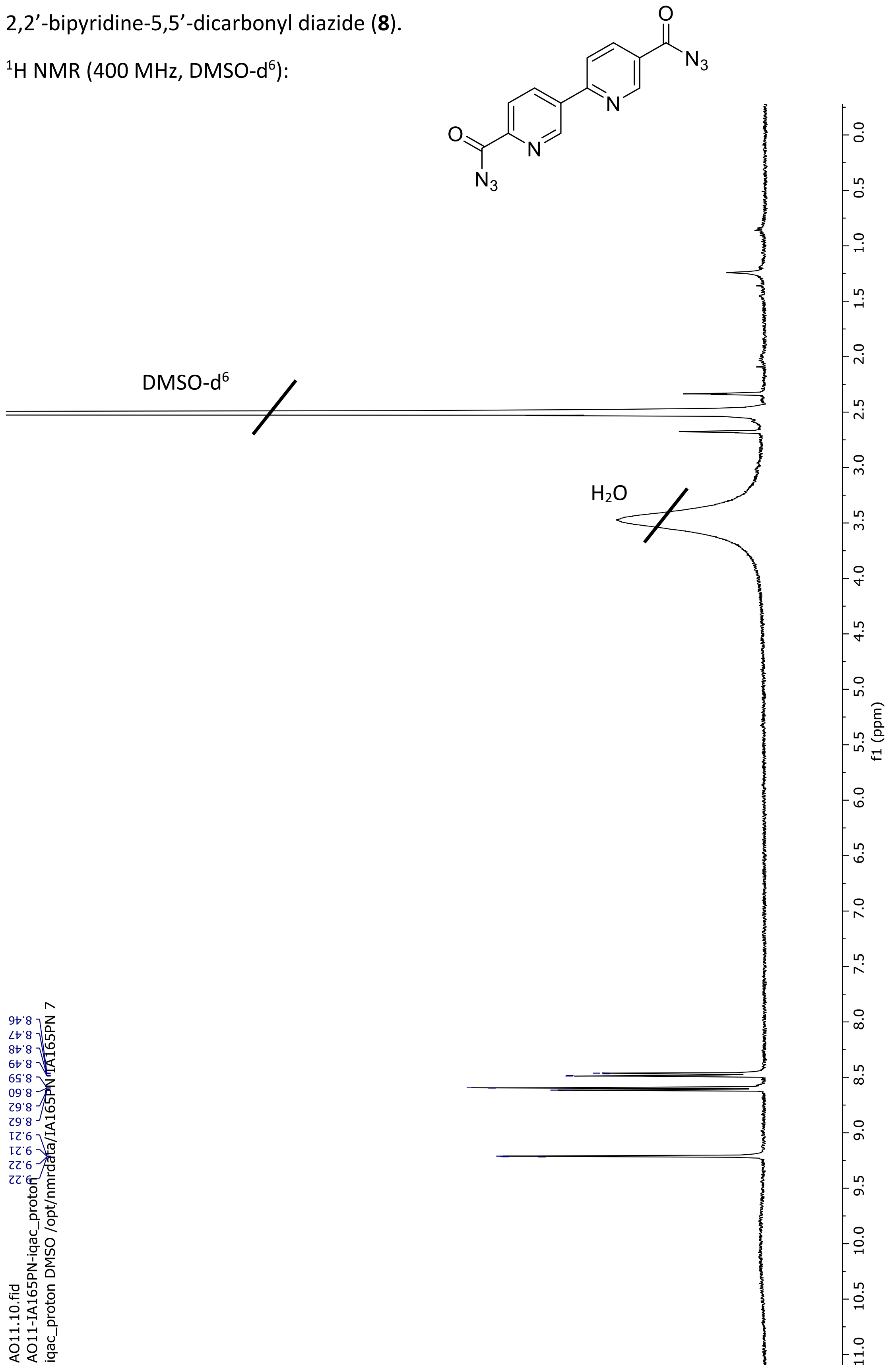

Figure S9. ^{1}H NMR spectrum of diacylazide **8**.

FTIR(ATR) of **8**:

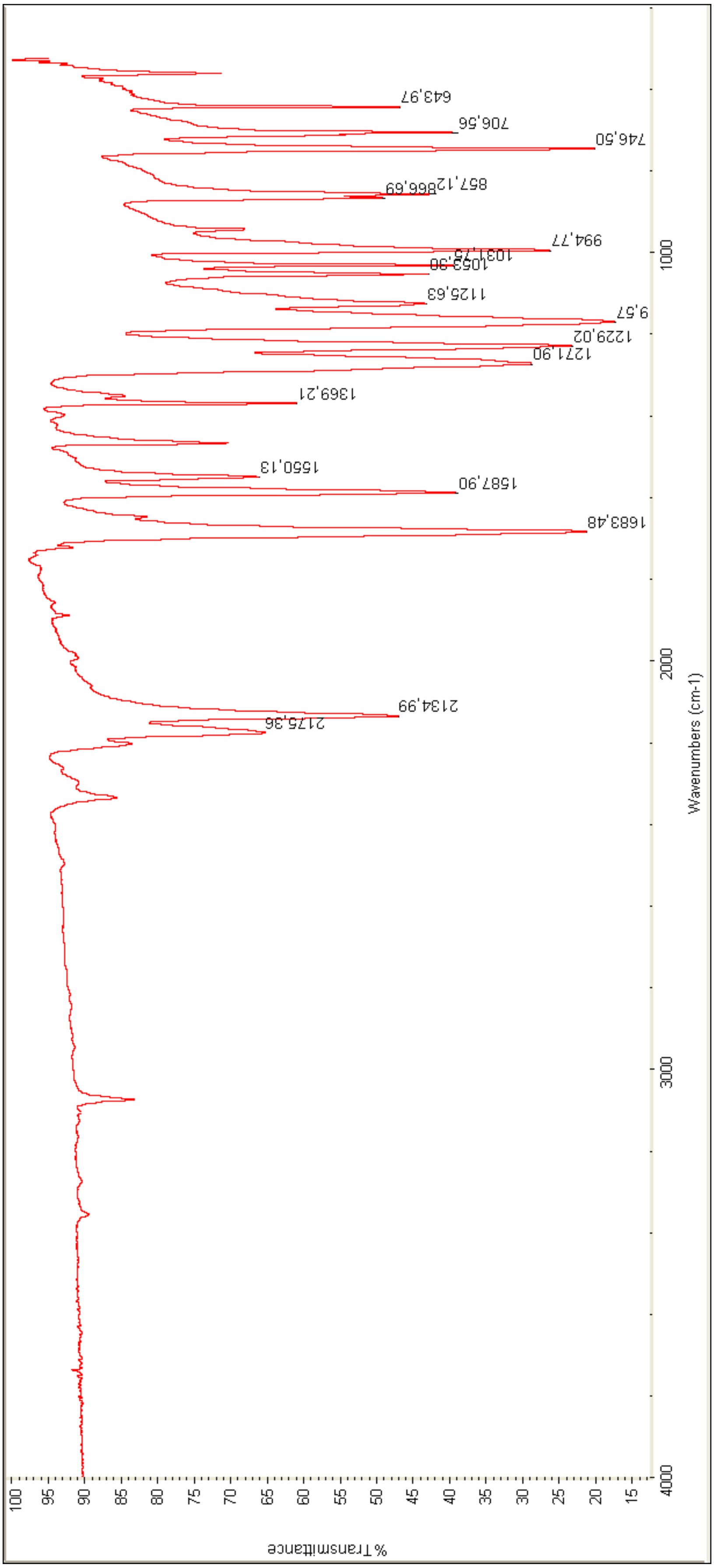

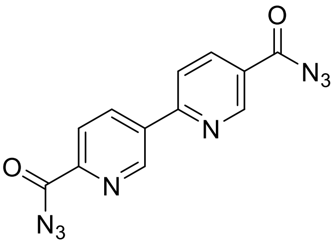

Figure S10. FTIR spectrum of diacylazide **8**.

Diethyl 2,2’-bipyridine-5,5’-diyldicarbamate (**7**):

^1H NMR (400 MHz, DMSO-d[6]) of **7**:

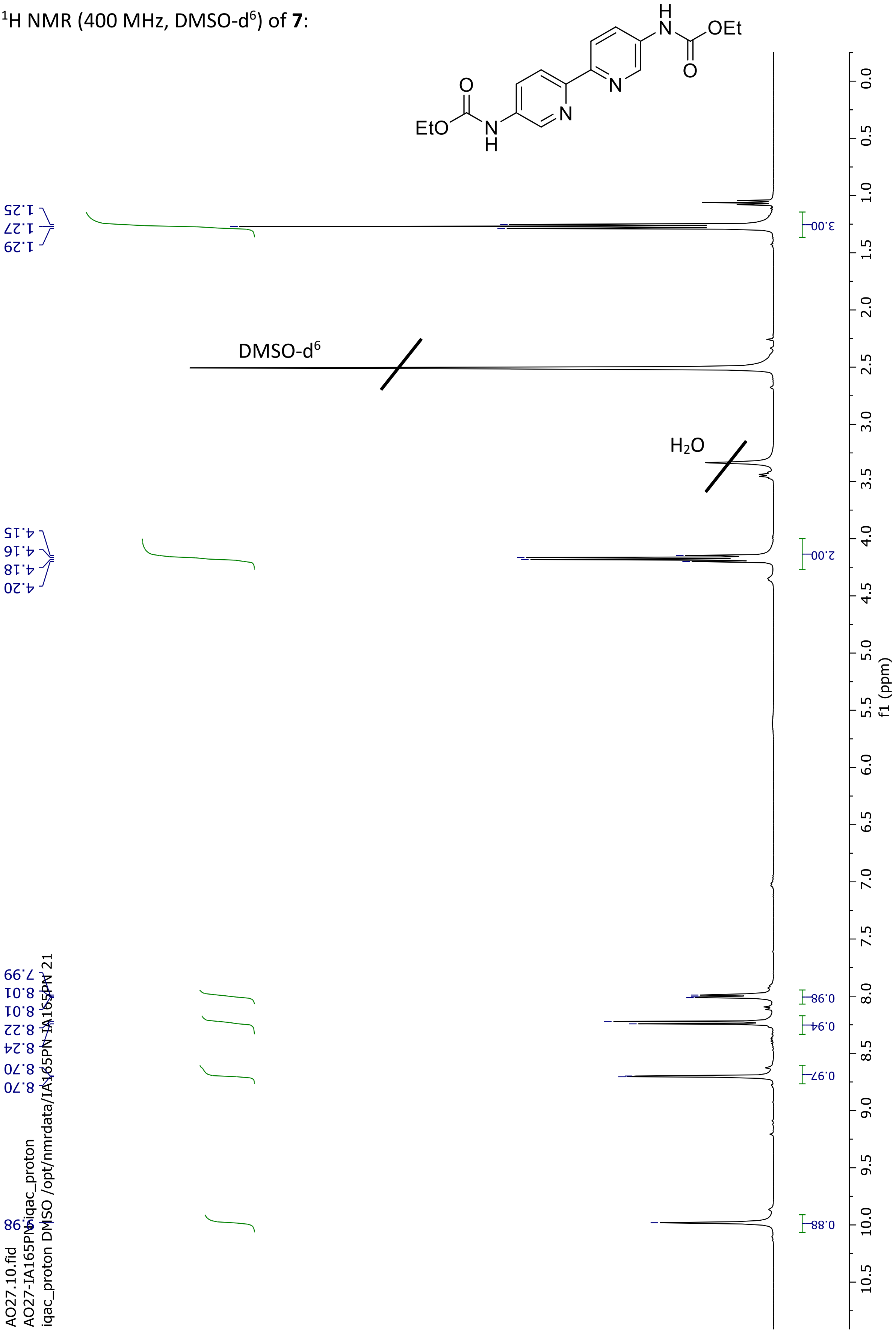

Figure S11. ^1H NMR spectrum of dicarbamate **7**.

2,2'-bipyridine-5,5'-diamine (9):

^{1}H NMR (400 MHz, DMSO-d[6]) of **9**:

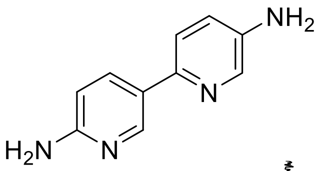

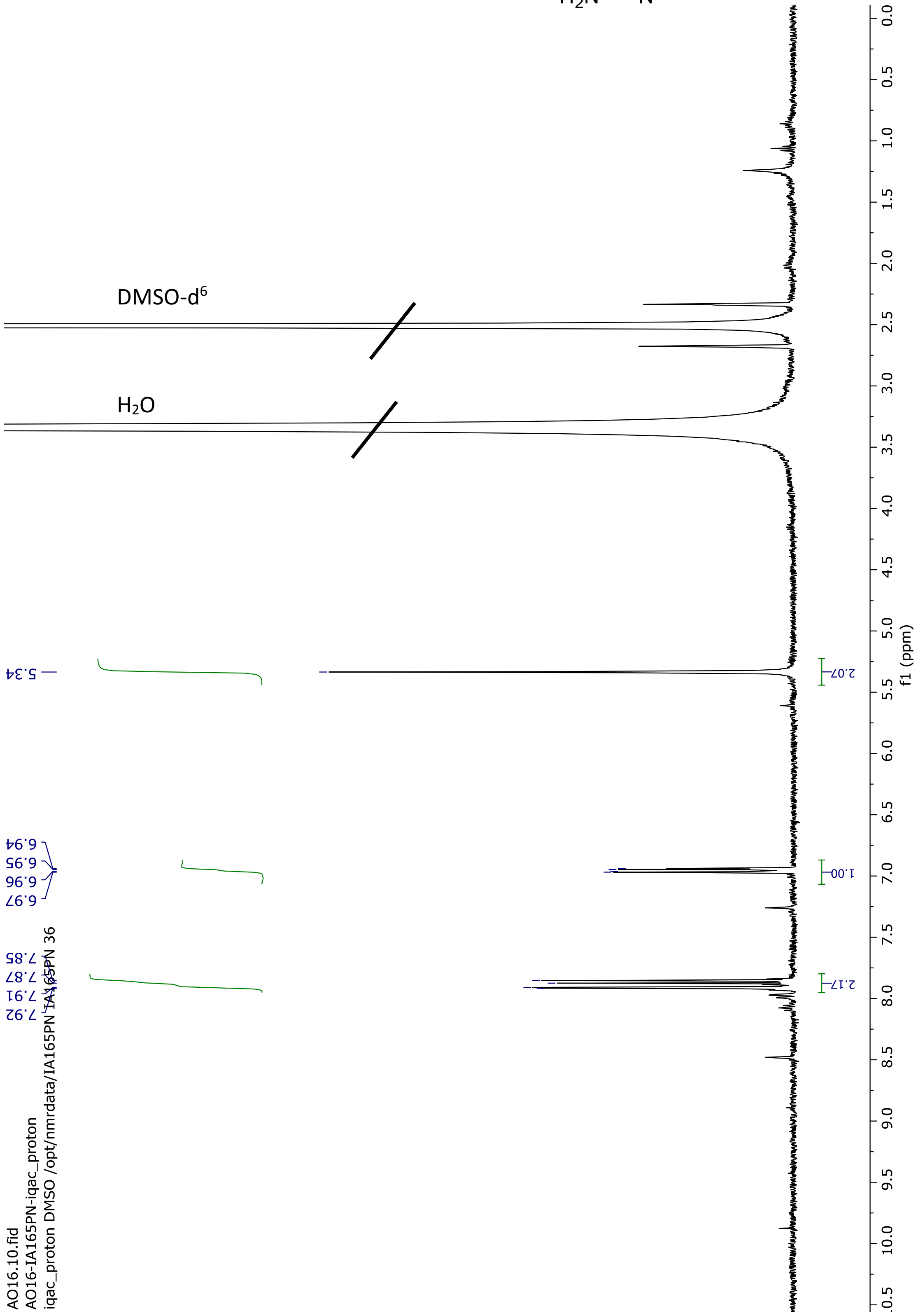

Figure S12. ^{1}H NMR spectrum of bipyridine diamine **9**.

5. <u>PROCEDURE FOR THE ASYMMETRIC ALDOL REACTION AND ee DETERMINATION</u> [4]

A mixture of the three catalyst components was stirred in 300 µL of dry THF and 10 µL of H_2O for 1 h. Then, 90 mg of *p*-nitrobenzaldehyde (0.595 mmol, 1 eq) and 617 µL of cyclohexanone (5.95 mmol, 10 eq) were added. When the reaction finished, H_2O was added and the solution was extracted with EtOAc. The organic phase was dried over anhydrous $MgSO_4$, filtered and the solvents evaporated in the rotavap. The crude product was purified by silica gel column chromatography using a gradient of Hexane (100 – 50 %) / EtOAc (0 – 50 %) to give 110 mg of the aldol product (74 % yield). Chiral analysis was performed using a Chiralpak IB column eluting with n-hexane / isopropanol (90/10) mobile phase, flow 1 mL/min, 30 minutes.

^{1}H-NMR (400 MHz, $CDCl_3$) δ 8.25 – 8.17 (m, 2H), 7.55 – 7.46 (m, 2H), 4.90 (d, *J* = 8.3 Hz, 1H), 4.31 – 3.75 (m, 1H), 2.59 (dddd, *J* = 13.9, 8.4, 5.5, 1.2 Hz, 1H), 2.54 – 2.45 (m, 1H), 2.36 (tdd, *J* = 13.6, 6.2, 1.2 Hz, 1H), 2.11 (ddt, *J* = 12.2, 5.8, 3.0 Hz, 1H), 1.91 – 1.80 (m, 1H), 1.67 (dt, *J* = 13.0, 3.8 Hz, 1H), 1.58 (ddt, *J* = 13.3, 10.0, 3.3 Hz, 2H), 1.45 – 1.31 (m, 1H) ppm.

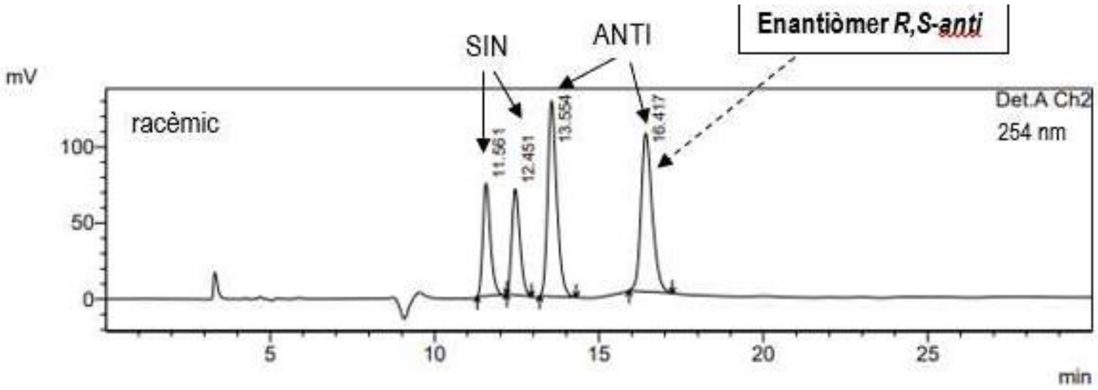

5 mol% Zn(TFA)$_2$+bipyPro$_2$+bipyHA$_2$ at rt:

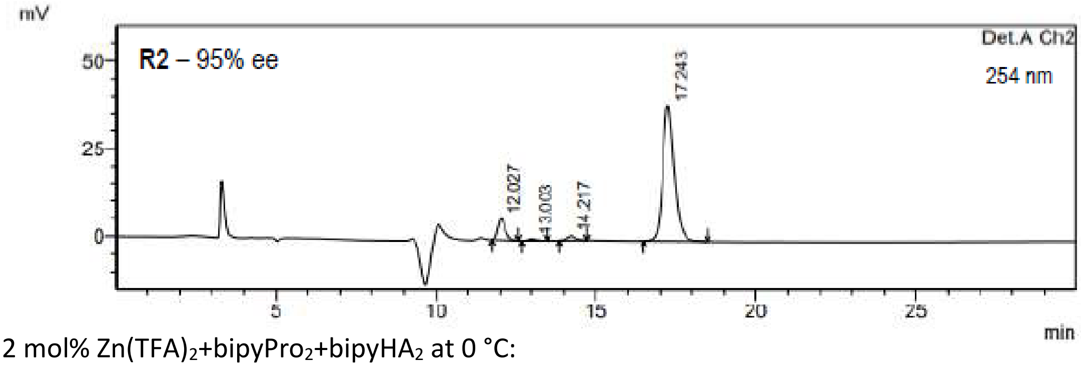

2 mol% Zn(TFA)$_2$+bipyPro$_2$+bipyHA$_2$ at 0 °C:

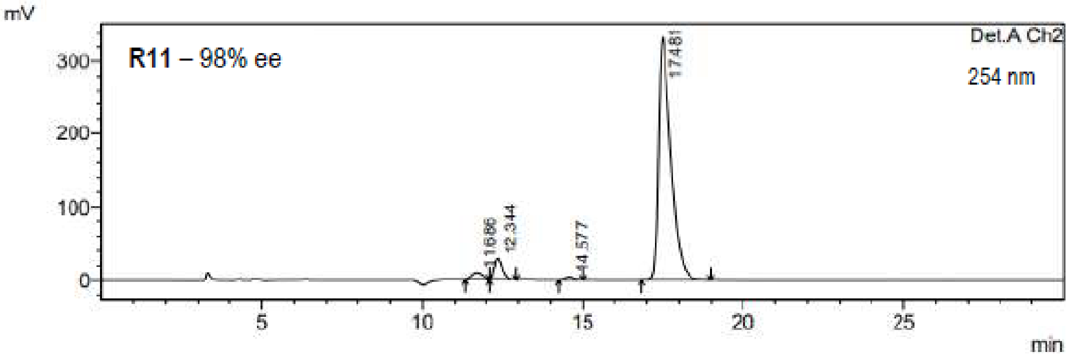

Figure S13. HPLC determination of enantiomeric excess.

For details and analyses on the other aldol products, see our previous publications.[4]

6. EFFECT OF SOLVENTS IN CONVERSION AND STEREOSELECTIVITY

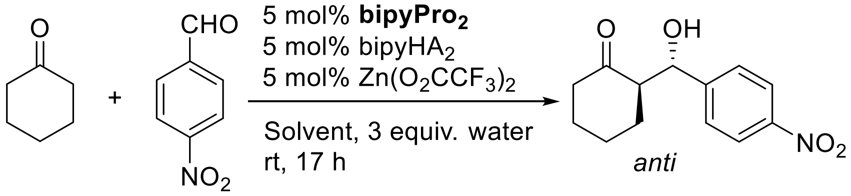

Entry	Solvent	Conv. [%]	d.r. (anti/syn)	ee [%]
1	THF	100	8/1	95
2	DMSO	92	4/1	83
3	DMF	98	5/1	92
4	ACN	74	5/1	94

Table S1. Effect of solvents in conversion and stereoselectivity.

7. DETERMINATION OF APARENT KINETIC CONSTANTS

Conversion vs. time plot for the aldol reaction between cyclohexanone and *p*-nitrobenzaldehyde, at rt. Fitting was performed with Origin software.

A) 5 mol% $Zn(TFA)_2$+**bipyPro$_2$**+**bipyHA$_2$**. k_{ap}= 1/t1 = 0.474 h^{-1}

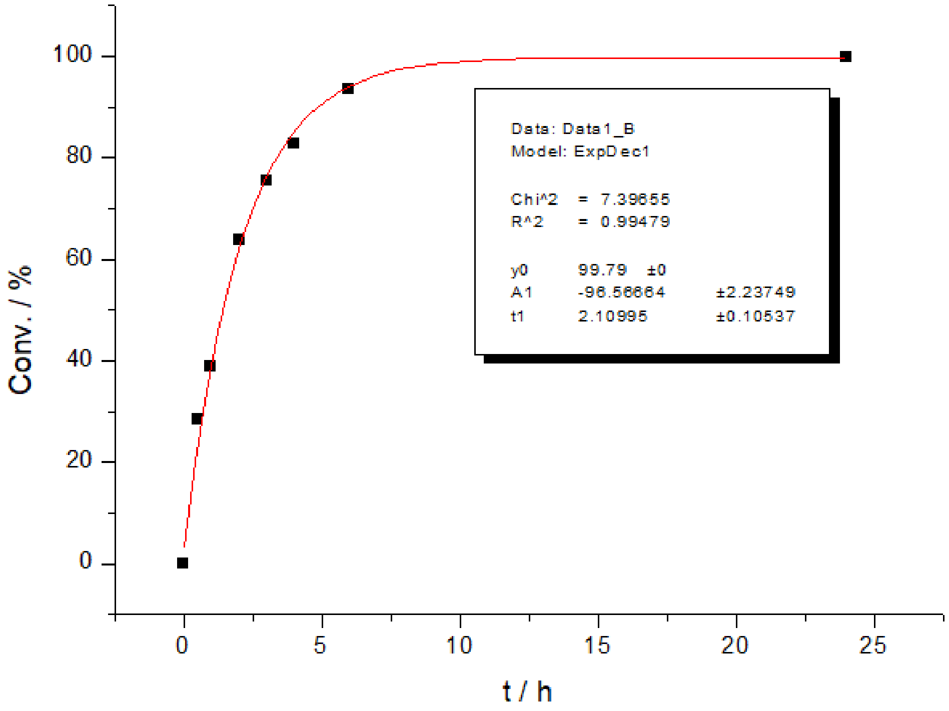

B) 5 mol% $Zn(TFA)_2$+**bipyPro$_2$.** k_{ap}= 1/t1 = 0.257 h^{-1}

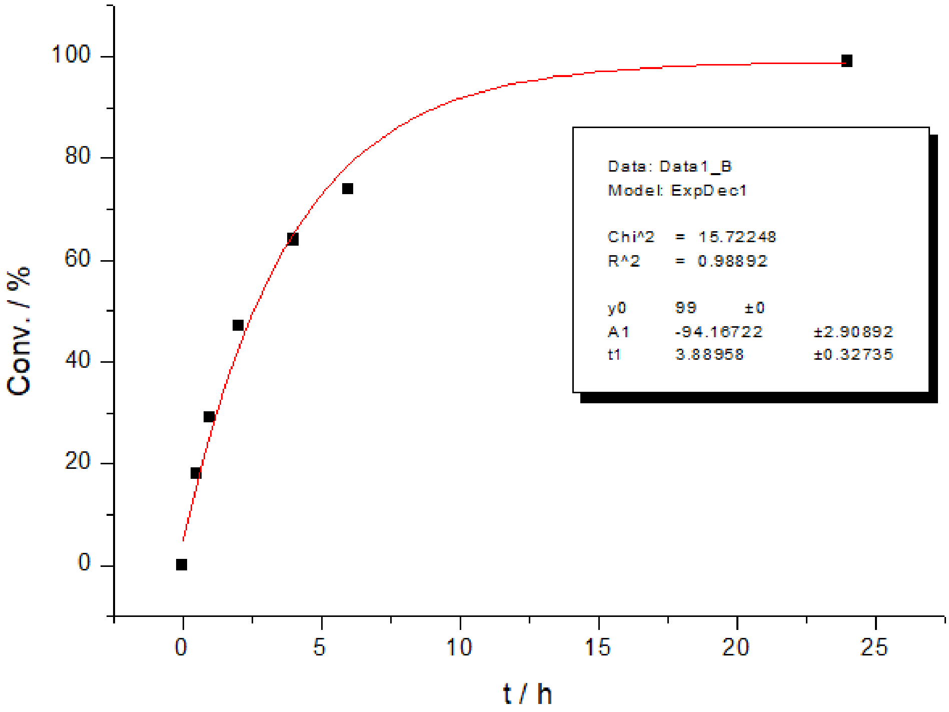

Figure S14. Conversion *vs*. time fitting and determination of apparent kinetic constants.

8. <u>DETERMINATION OF CATALYST ORDER </u>[5]

The graphical procedure developed by J. Burés was used.[5] Reaction of cylclohexanone and *p*-nitrobenzaldehyde at rt with different catalyst loadings (1, 2 and 5 mol% of $Zn(TFA)_2$+**bipyPro$_2$**+**bipyHA$_2$**)) was sampled and conversion determined by ^{1}H NMR. From there, [*p*-nitrobenzaldehyde] vs. time plots were constructed:

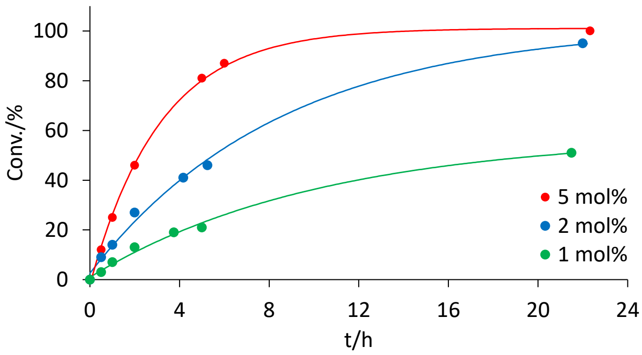

Graphical fitting of different options concluded that the order of catalyst was 1:

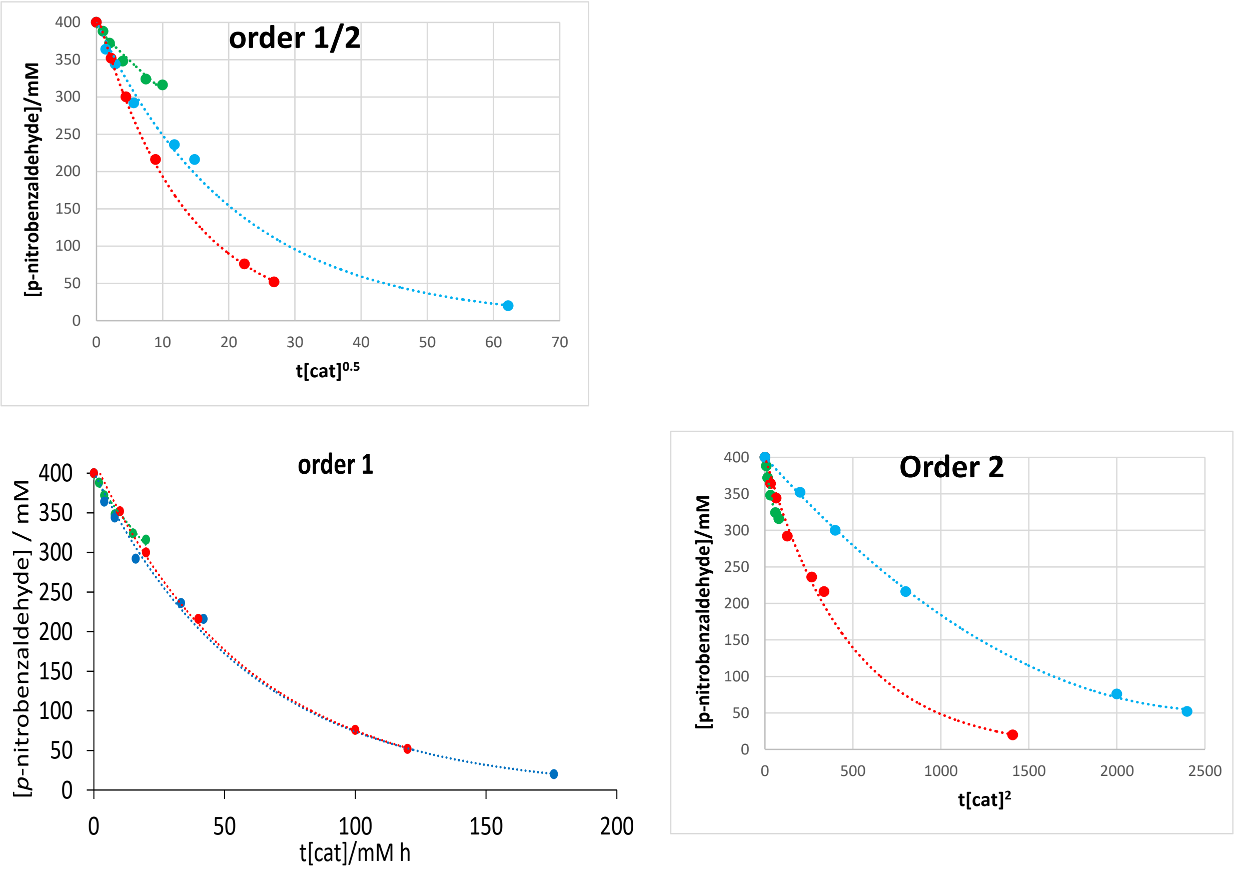

Figure S15. Determination of catalyst order for $Zn(TFA)_2$+**bipyPro$_2$**+**bipyHA$_2$**.

Since three different zinc complexes with two ligands can arise from a 1:1:1 mixture of Zn:**bipyPro$_2$**:**bipyHA$_2$**, ranging from the statistical 25:50:25 distribution to 100% Zn(**bipyPro$_2$**)(**bipyHA$_2$**), the validity of the catalyst order calculation could be challenged.

However, firstly, we had found that the equilibrium constant formation for our previous bipyridine based ZnL1L2 complex was $3.5x10^{11}$ M^{-1}, whereas the $Zn(L1)_2$ and $Zn(L2)_2$ equilibrium constants were $4x10^{10}$ and $1x10^{11}$ M^{-1}, in line with the literature. These values led to the predominant formation of 71% ZnL1L2, and 14% of $Zn(L1)_2$ and $Zn(L2)_2$ each, overcoming the statistical distribution.[4c]

Secondly, as a consequence of these large formation constants the zinc complexes distribution does not change, whatever it is, at least in the range of concentrations used for catalysis (from 4 to 20 mM). This is, the different zinc complexes are not under real reversible equilibrium. See Figure S16 below.

Finally, since the catalyst order determination is therefore not affected by the real concentration of catalyst, which remains proportional to the amount introduced initially into the reaction, we have used for the catalyst order determination the weighed molar amount of the different components.

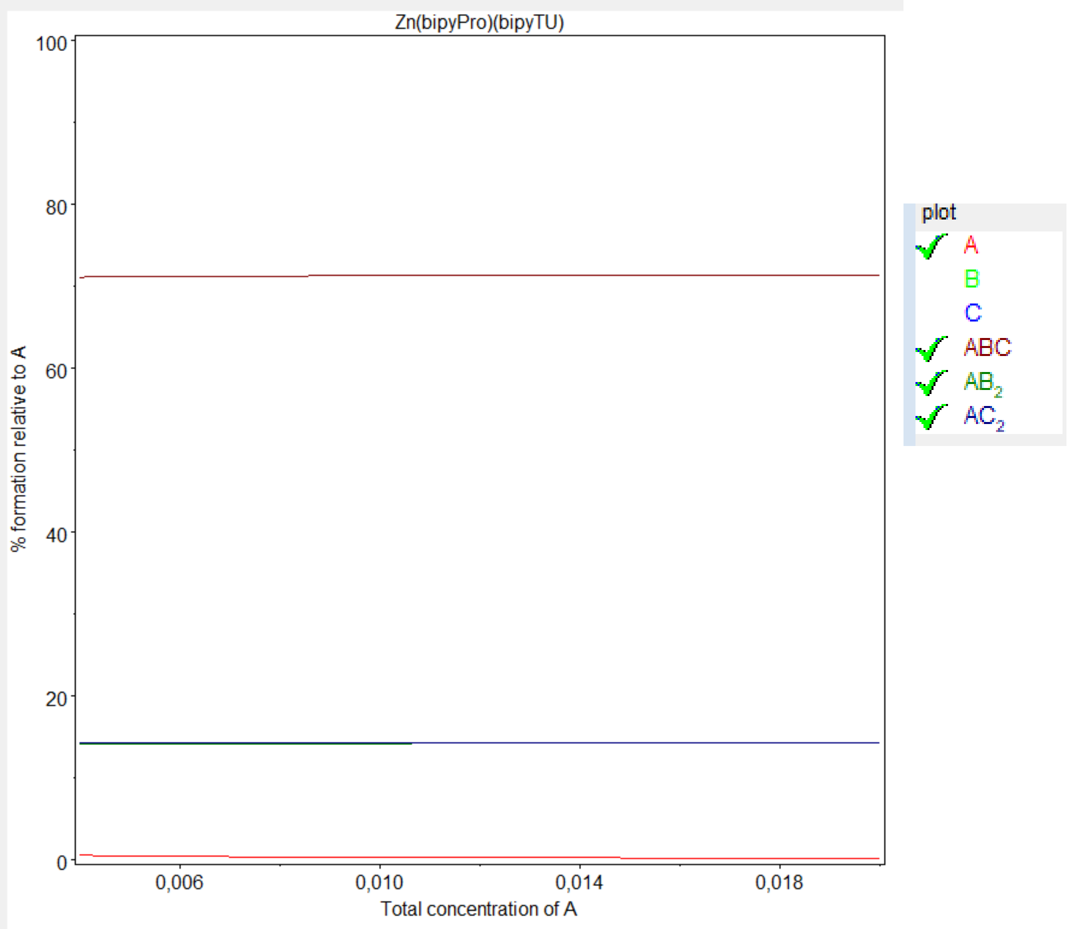

Figure S16. Species distribution for a Zn complex with two bipyridine ligands in the range 4 to 20 mM with K_{eq} = $3.5x10^{11}$ M^{-1} (Zn(**bipyPro**)(**bipyTU**); ABC),$4x10^{10}$ M^{-1} (Zn(**bipyPro$_2$**); AB_2) and $1x10^{11}$ M^{-1} (Zn(**bipyTU**)$_2$; AC_2). Plotted from data in ref. 4c using HySS software.

9. DETERMINATION OF CATALYST TOF

First, NMR conversion vs. time data was obtained through sampling the corresponding aldol reactions between cyclohexanone and *p*-nitrobenzaldehyde at rt, using 5 mol% of both catalytic systems ($Zn(TFA)_2$+**bipyPro**+**bipyTU** [old system][4c] vs. $Zn(TFA)_2$+**bipyPro₂**+**bipyHA₂** [new system]). Then, a [*p*-nitrobenzaldehyde] vs. time plot was constructed. Data points were fitted to polynomial equations:

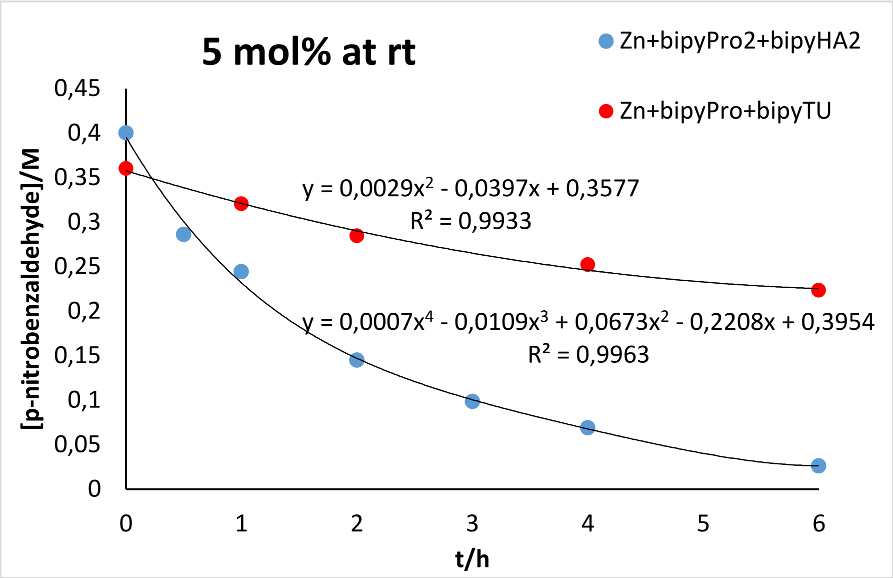

Figure S17. Fitting curves for [*p*-nitrobenzaldehyde] vs. time for the current (blue dots) and previous (red dots) catalytic systems.

These equations were derived to obtain rate equations, which furnished the TOF for each catalyst upon division by the ligand (**bipyPro** or **bipyPro₂**) concentration:

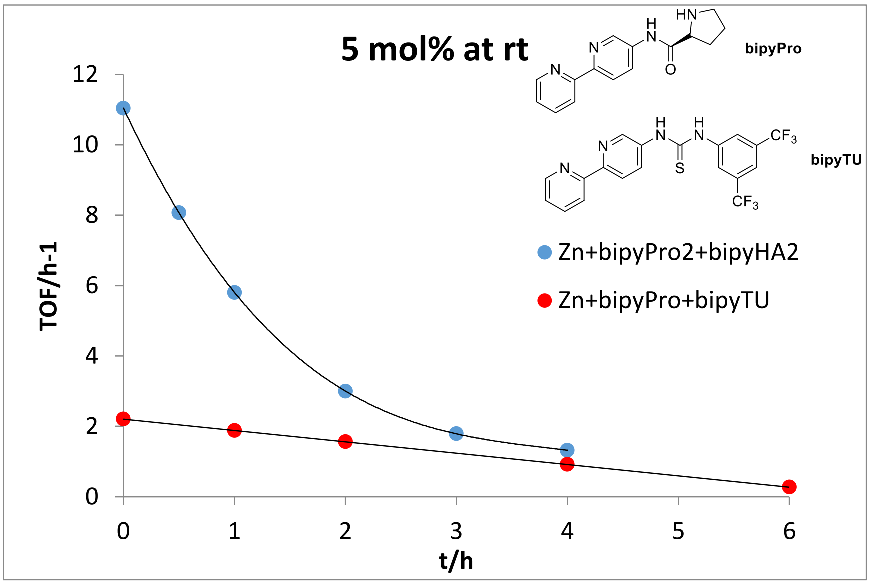

Figure S18. Calculated TOF *vs*. time curves for the current (blue dots) and previous (red dots) catalytic systems.

From this plot, intitial TOF's were found:

TOF_0[**bipyPro₂**] = 11 h^{-1}

TOF_0[**bipyPro**] = 2.2 h^{-1}

For comparison, since **bipyPro$_2$** has double the catalytic sites than **bipyPro**, TOF_0[**bipyPro$_2$**] must be divided by 2 (11 / 2 = 5.5 h^{-1} *vs*. 2.2 h^{-1} from **bipyPro**), or alternatively, TOF_0[**bipyPro**] must be multiplied by 2 (2.2 x 2 = 4.4 h^{-1} *vs*. 11 h^{-1} from **bipyPro$_2$**). In any case, the catalytic system containing **bipyPro$_2$** is significantly faster. Indeed, the system $Zn(TFA)_2$+**bipyPro$_2$**+**bipyHA$_2$** is 5 times faster, not twice faster as it could be expected by doubling the number of catalytic sites in the ligand.

10. PROPOSED REACTION MECHANISM AND ORIGIN OF STEREOSELECTIVITY

This proposed mechanism must be understood as based on current and previous research of our group.[4c] Please, keep in mind that it is a speculative mechanism lacking comprehensive kinetic and theoretical studies. It is presented herein to give the vision of the authors on the catalyst operation (Please, check also Sections 8 and 9 in this Supporting Information).
We propose a working mechanism in which a predominant zinc-templated bifunctional catalyst behaves as the catalytically most active species.
The Zn(**bipyPro₂**)(**bipyHA₂**) complex would work *via* an enamine mechanism with cyclohexanone using one prolinamide moiety and attract the aldehyde through hydrogen bonding through one of the hydrazide groups. In this way the asymmetric aldol reaction could take place in a stereocontrolled fashion. The observed diastereo- and enantioselectivity can be rationalized in this way too.

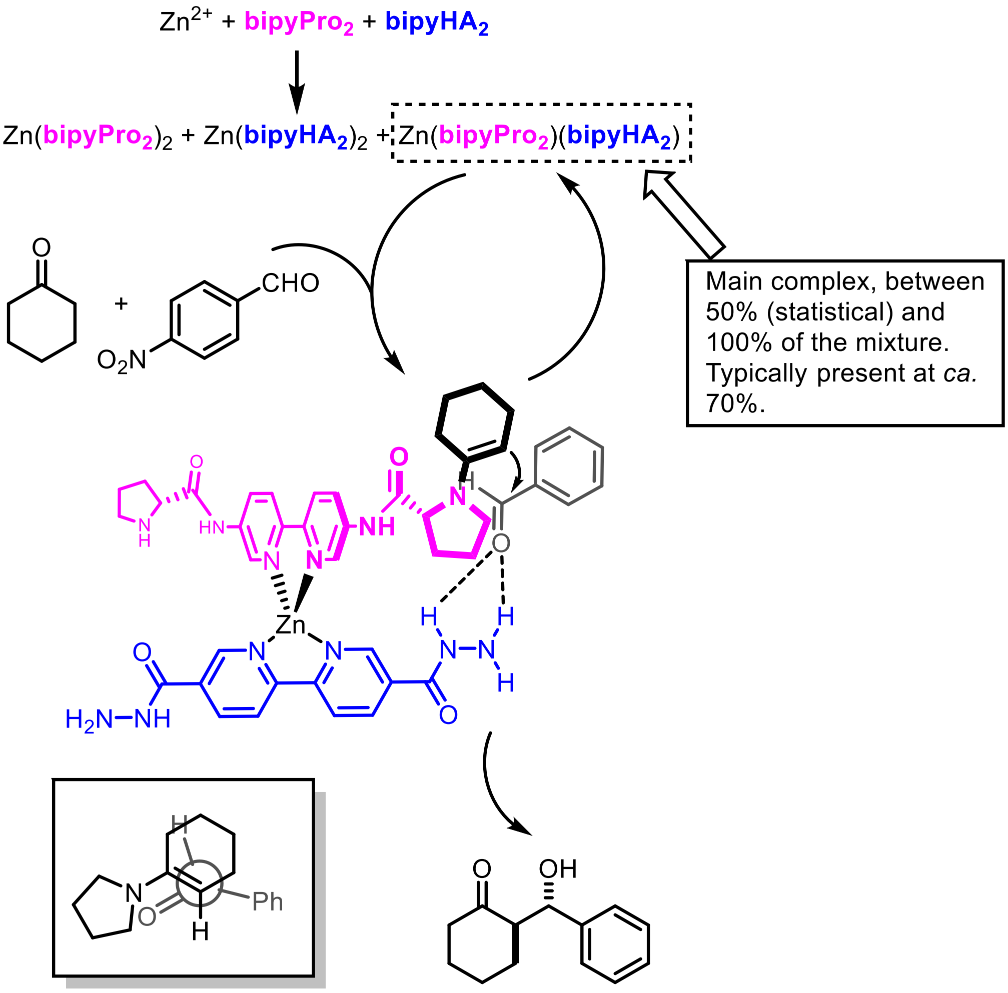

Figure S19. Simplified mechanistic proposal and model for the observed stereoselectivity.

12. TRANSITION STATE MODEL FIGURES

(A)

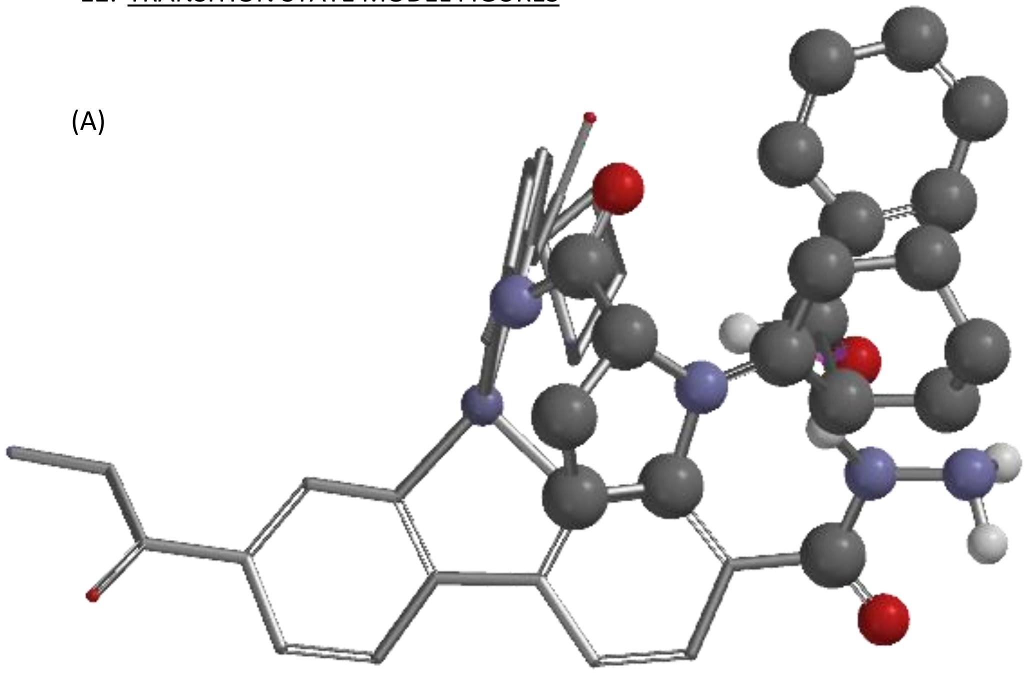

(B)

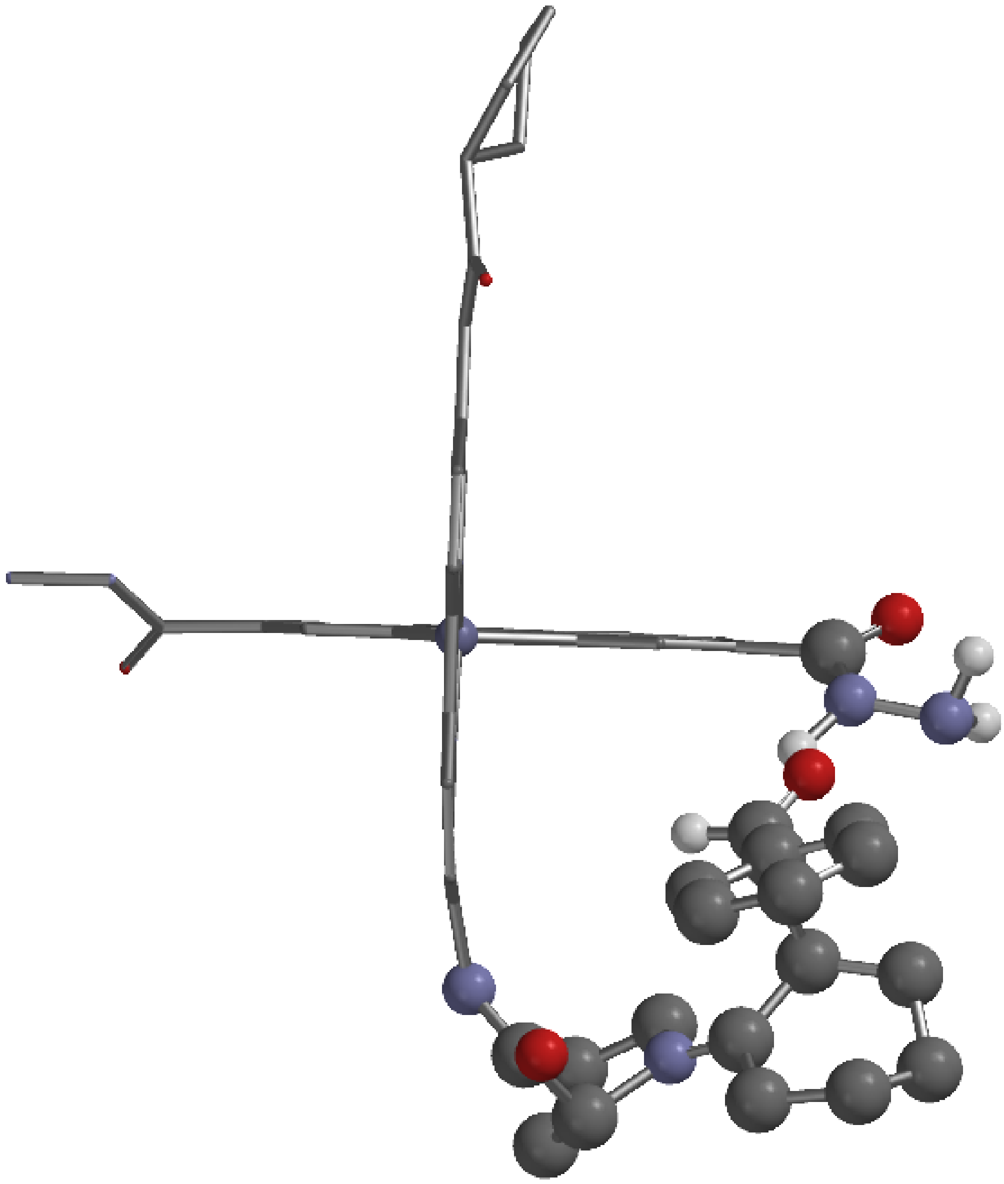

(C)

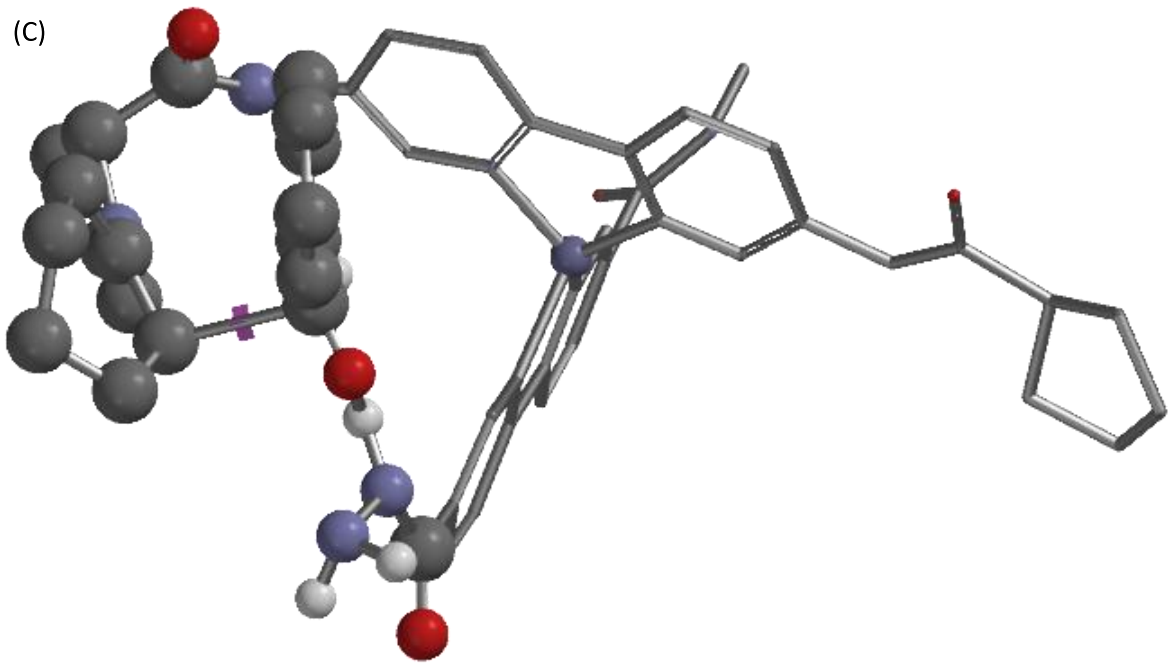

(D)

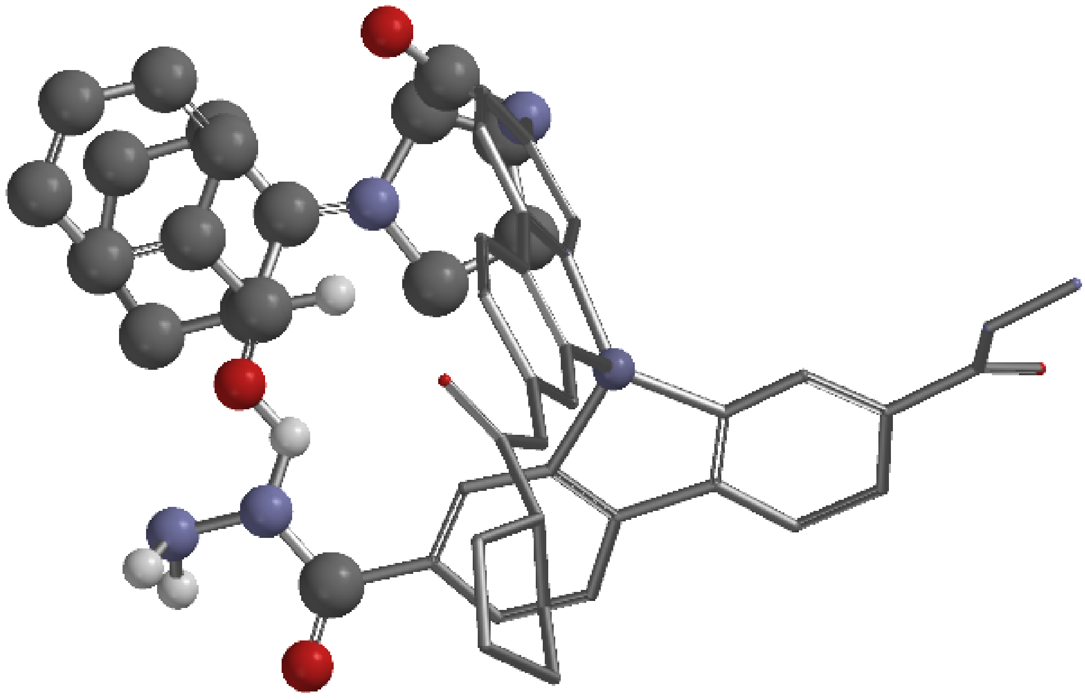

(E)

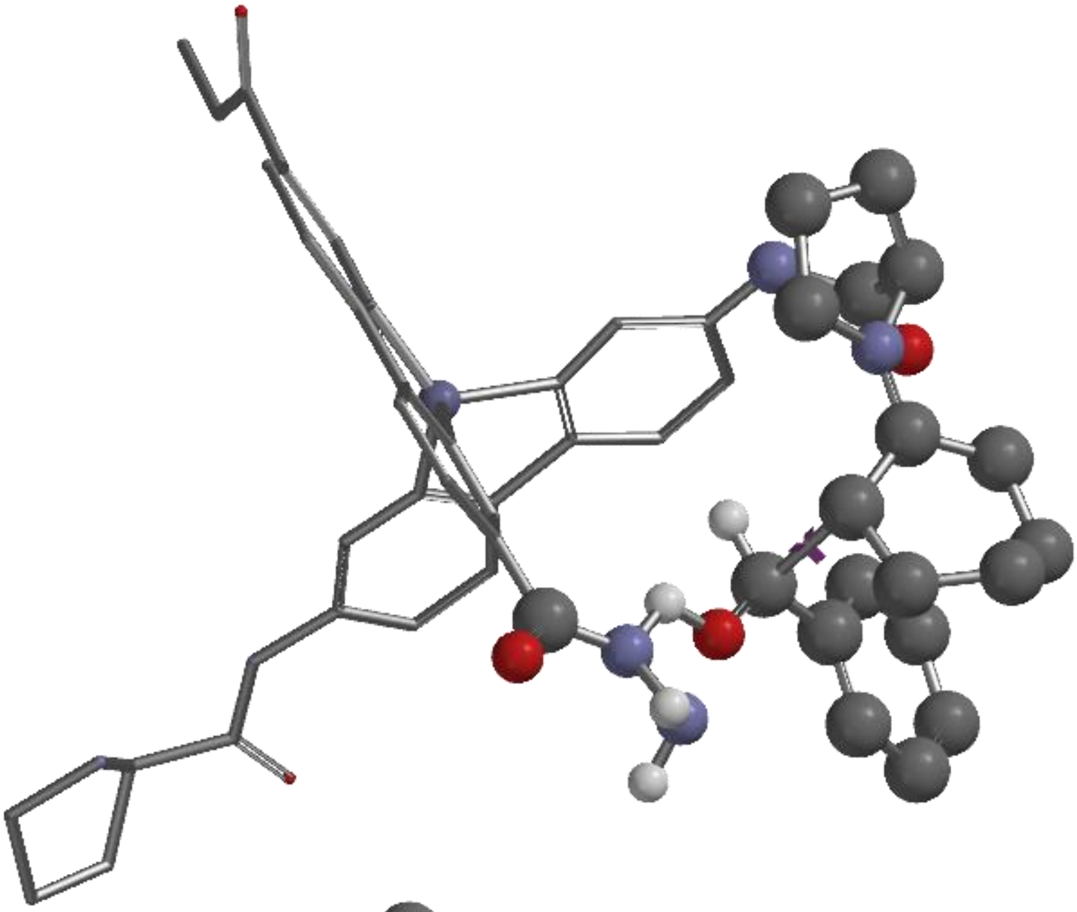

(F)

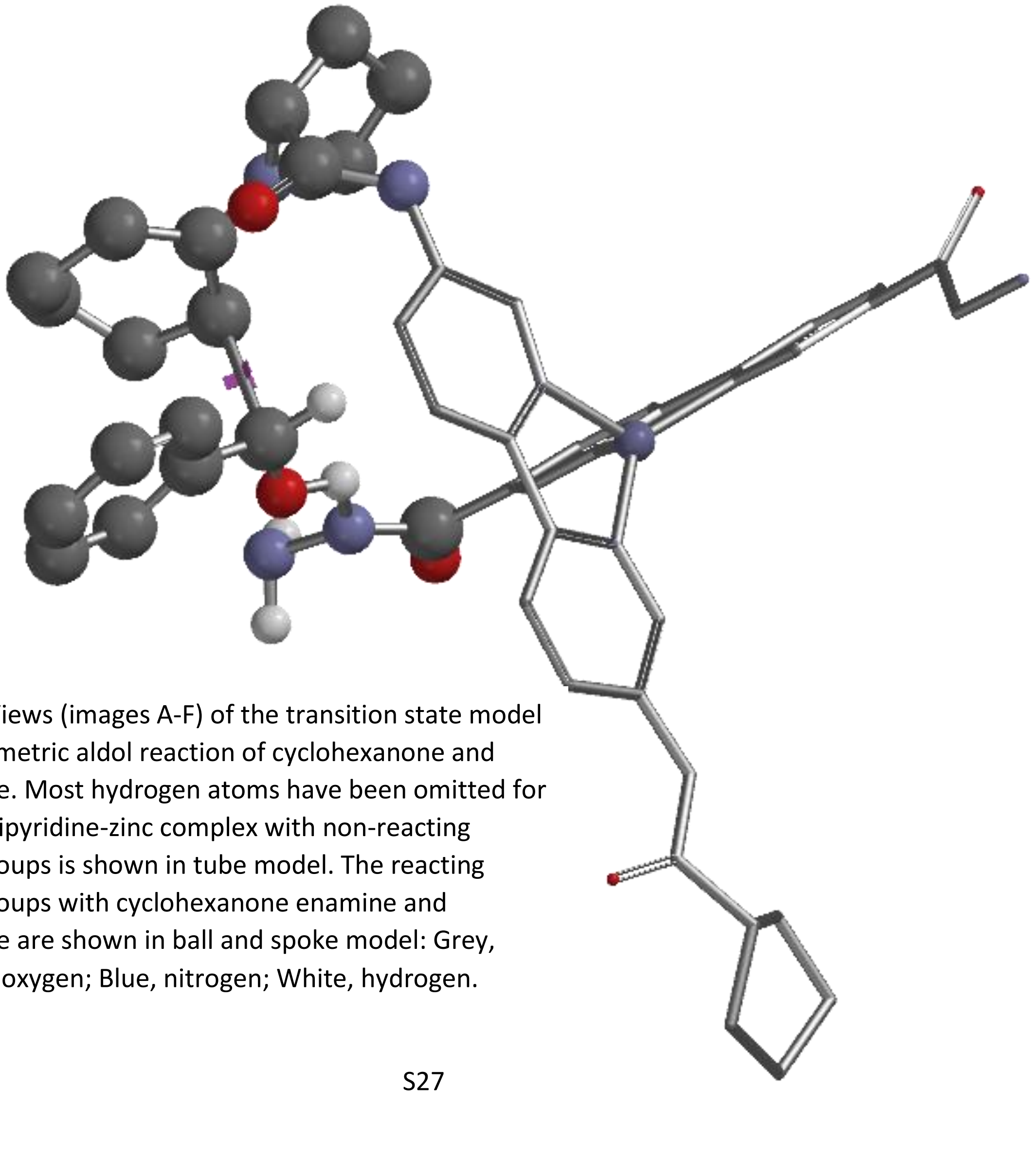

Figure S20. Views (images A-F) of the transition state model for the asymmetric aldol reaction of cyclohexanone and benzaldehyde. Most hydrogen atoms have been omitted for clarity. The bipyridine-zinc complex with non-reacting functional groups is shown in tube model. The reacting functional groups with cyclohexanone enamine and benzaldehyde are shown in ball and spoke model: Grey, carbon; Red, oxygen; Blue, nitrogen; White, hydrogen.

13. REFERENCES

[1] Z. Yang, M. Shen, M. Tang, W. Zhang, X. Cui, Z. Zhang, H. Pei, Y. Li, M. Hu, P. Bai, L. Chen. Discovery of 1,2,4-oxadiazole-Containing hydroxamic acid derivatives as histone deacetylase inhibitors potential application in cancer therapy. *Eur. J. Med. Chem.* **2019**, *178*, 116-130

[2] S. Saha, S. Santra, P. Ghosh. Cu^{II}-Templated Threading of a Bis-amide-tris-amine Macrocycle by Substituted 2,2′-Bipyridyl Derivatives Assisted by Strong π–π Stacking and Second-Sphere H-Bonding Interactions. *Eur. J. Inorg. Chem.* **2014**, 2029-2037.

[3] (a) Janiak, C.; Deblon, S.; Wu, H.-P. Syntheses of 5,5′-Disubstituted 2,2′-Bipyridines. *Synth. Commun.*, **1999**, *29*, 3341-3352. (b) Janiak, C.; Deblon, S.; Wu, H.-P.; Kolm, M. J.; Klufers, P.; Piotrowski, H.; Mayer, P. Modified Bipyridines: 5,5′-Diamino-2,2′-bipyridine Metal Complexes Assembled into Multidimensional Networks via Hydrogen Bonding and π–π Stacking Interactions. *Eur. J. Inorg. Chem.*, **1999**, 1507-1521

[4] (a) A. Serra-Pont, I. Alfonso, C. Jimeno and J. Solà. Dynamic assembly of a zinc-templated bifunctional organocatalyst in the presence of water for the asymmetric aldol reaction. *Chem. Commun*., **2015**, *51*, 17386-17389. (b) A. Serra-Pont, I. Alfonso, J. Solà and C. Jimeno. A copper-templated, bifunctional organocatalyst: a strongly cooperative dynamic system for the aldol reaction. *Org. Biomol. Chem*., **2017**, *15*, 6584-6591. (c) A. Serra-Pont, I. Alfonso, J. Solà and C. Jimeno. An efficient dynamic asymmetric catalytic system within a zinc-templated network. *Chem. Commun*., **2019**, 55, 7970-7973. (d) A. M. Valdivielso, A. Catot, I. Alfonso and C. Jimeno. Intramolecular hydrogen bonding guides a cationic amphiphilic organocatalyst to highly stereoselective aldol reactions in water. *RSC Adv*., **2015**, *5*, 62331-62335.

[5] (a) J. Burés. A Simple Graphical Method to Determine the Order in Catalyst. *Angew. Chem. Int. Ed.*, **2016**, *55*, 2028-2031. (b) C. D. T. Nielsen and J. Burés. Visual kinetic analysis. *Chem. Sci.*, **2019**, *10*, 348-353
